# Supplementary material for: Pulmonary sarcoidosis with and without extrapulmonary involvement: a cross-sectional and observational study in China
Source: BMJ Open. 2018 Feb 16;8(2):e018865. doi: 10.1136/bmjopen-2017-018865 (PMC5829779; doi:10.1136/bmjopen-2017-018865)
Supplement: Author's manuscript [file bmjopen-2017-018865.draft_revisions.pdf]

BMJ Open is committed to open peer review. As part of this commitment we make the peer review history of every article we publish publicly available.

When an article is published we post the peer reviewers' comments and the authors' responses online. We also post the versions of the paper that were used during peer review. These are the versions that the peer review comments apply to.

The versions of the paper that follow are the versions that were submitted during the peer review process. They are not the versions of record or the final published versions. They should not be cited or distributed as the published version of this manuscript.

BMJ Open is an open access journal and the full, final, typeset and author-corrected version of record of the manuscript is available on our site with no access controls, subscription charges or pay-per-view fees (http://bmjopen.bmj.com).

If you have any questions on BMJ Open's open peer review process please email info.bmjopen@bmj.com

BMJ Open

Comparison of clinical characteristics and prognosis between Chinese pulmonary sarcoidosis patients with and without extrapulmonary involvement

Journal:	*BMJ Open*
Manuscript ID	bmjopen-2017-018865
Article Type:	Research
Date Submitted by the Author:	27-Jul-2017
Complete List of Authors:	li, chengwei; Shanghai Pulmonary Hospital, Tongji University School of Medicine, Department of Respiratory and Critical Care Medicine<br>tao, rujia; Shanghai Pulmonary Hospital, Tongji University School of Medicine, Department of Respiratory and Critical Care Medicine<br>zou, danfeng; Shanghai Pulmonary Hospital, Tongji University School of Medicine, Clinic and Research Center of Tuberculosis, Shanghai Key Lab of Tuberculosis<br>li, manhui; Shanghai Pulmonary Hospital, Tongji University School of Medicine, Department of Respiratory and Critical Care Medicine<br>xu, xin; Shanghai Pulmonary Hospital, Tongji University School of Medicine, Department of Respiratory and Critical Care Medicine<br>cao, weijun; Tongji University Affiliated Shanghai Pulmonary Hospital, Department of Respiratory Medicine
**Primary Subject Heading**:	Respiratory medicine
Secondary Subject Heading:	Respiratory medicine, Radiology and imaging
Keywords:	sarcoidosis, extrapulmonary involvement, fatigue, prognosis

SCHOLARONE™
Manuscripts

Comparison of clinical characteristics and prognosis between Chinese pulmonary sarcoidosis patients with and without extrapulmonary involvement

Cheng-Wei Li[a#], Ru-Jia Tao[b#], Dan-Feng Zou[c#], Man-Hui Li[b], Xin Xu[b], Wei-Jun Cao[b]

Institution:
a: Department of Respiratory and Critical Care Medicine, Shanghai Pulmonary Hospital, Soochow University, Suzhou 215006, China
b: Department of Respiratory and Critical Care Medicine, Shanghai Pulmonary Hospital, Tongji University School of Medicine, Shanghai 200433, China
c: Clinic and Research Center of Tuberculosis, Shanghai Key Lab of Tuberculosis, Shanghai Pulmonary Hospital, Tongji University School of Medicine, Shanghai 200433, China
#: These authors contributed equally.

Corresponding author: Wei-Jun Cao, Department of Respiratory and Critical Care Medicine, Shanghai Pulmonary Hospital, Tongji University School of Medicine, No. 507 Zhengmin Road, Shanghai, 200433, China. E-mail: weijuncao@126.com

Word count: 2,154 words.

For peer review only

ABSTRACT

Objectives: Sarcoidosis is a multisystem disease characterized by the formation of granulomas within various organs, mainly the lungs. Several studies from different countries have been undertaken to investigate sarcoidosis with extrapulmonary involvement but from China. The objective of this study is to investigate a comparative clinical analysis in Chinese sarcoidosis patients with and without extrapulmonary involvement.

Methods: Data from inpatients diagnosed with sarcoidosis at Shanghai Pulmonary Hospital (Shanghai, China) between January 2009 and December 2014 were retrospectively collected and analyzed. Six hundred and thirty-six biopsy proven sarcoidosis patients were included in the study, including 378 isolated pulmonary sarcoidosis and 258 pulmonary sarcoidosis plus extrapulmonary involvement.

Results: Two hundred and fifty eight (40.6%) pulmonary sarcoidosis patients had extrapulmonary involvement. Extrapulmonary localizations were detected mostly in extrathoracic lymph nodes (n=147) and skin (n=86). Statistically significant differences were demonstrated between patients with pulmonary plus extrapulmonary involvement and patients with isolated pulmonary sarcoidosis for fatigue (16.6% vs. 8.3%, $p<0.05$), serum angiotensin-converting enzyme (SACE) levels (79.0±46.9 IU/L vs. 69.7±38.7 IU/L, $p<0.05$), and high-resolution computed tomography (HRCT) findings (53.8% vs. 46.2%, $p<0.05$).

Conclusions:Extrapulmonary involvement was common in Chinese sarcoidosis patients, with the most common sites being extrathoracic lymph nodes and skin. Sarcoidosis patients with extrapulmonary involvement were more symptomatic (fatigue), had higher SACE levels and more deteriorating HRCT findings, to which clinicians should pay attention.

Keywords: sarcoidosis; extrapulmonary involvement; fatigue; prognosis

For peer review only

Article summary

Strengths and limitations of this study

1. The present study provides a large number of Chinese sarcoidosis patients with and without extrapulmonary involvement.
2. The sarcoidosis patients had a long term followe-up (24-42 months) on chest HRCT.
3. There is a risk of selection bias due to the retrospective design.

For peer review only

INTRODUCTION

Sarcoidosis is a granulomatous disease of unknown etilogy. It is characterized by the formation of immune granulomas in many involved organs.[1,2] Sarcoidosis is a worldwide disease, with its prevalence ranging from 4.7 to 64 in 100 000, and its incidence from 1.0 to 35.5 in 100 000 per year.[3] However, few epidemiological data in China is not available.

Sarcoidosis is a multisystem disease. Although the lungs are most commonly involved, pulmonary sarcoidosis is often accompanied by the involvement of extrapulmonary organs,[1,4] such as the skin, eyes, and heart that are significantly affected.[5] A Case Control Etiologic Study of Sarcoidosis (ACCESS) showed that among 736 patients with sarcoidosis, 95% had pulmonary involvement, and 368 out of these 736 patients (50%) had concomitant extrapulmonary involvement.[6] Extrapulmonary involvement can be the major presentation of the disease and sometimes may be life-threatening.[5,7] Moreover, the assessment of pulmonary and extrapulmonary organ involvement is important for clinical treatment decisions.[8]

It is well known that the features of sarcoidosis have varied widely among various populations around the world. To date, several studies from different countries have been undertaken to investigate sarcoidosis with extrapulmonary involvement. However, there is limited information in Chinese sarcoidosis patients. Therefore, the purpose of this study is to compare the clinical characteristics and prognosis of Chinese pulmonary sarcoidosis patients with and without extrapulmonary involvement.

METHODS

Data were retrospectively collected from inpatients diagnosed with sarcoidosis at the Shanghai Pulmonary Hospital (Shanghai, China) between January 2009 and December 2014. Patients who had not received a chest radiograph or chest high resolution computed tomography (HRCT) scan examination or who had not received tissue biopsy were excluded. All aspects of the study were performed in accordance with relevant guidelines and regulations.

Diagnosis of sarcoidosis is based on standardized criteria,[1] which detailed the features of sarcoidosis, including clinical and radiological presentation, and pathological findings (noncaseating epithelioid granulomas), as well as evidence of no alternative diseases (infections, particularly tuberculosis; occupationally induced, environmentally induced, and drug-induced granulomatosis; common variable immune deficiency; Blau's syndrome; sarcoid-like reactions in cancers and lymphomas, and other idiopathic granulomatosis).[3]

Organ involvement was determined and classified in each patient in accordance with criteria proposed in the ACCESS formulation,[9] based on findings from history, physical examination, and laboratory testing. According to the organs affected by sarcoidosis, patients were divided into two groups: isolated pulmonary sarcoidosis and pulmonary sarcoidosis with extrapulmonary involvement.

The following variables were obtained from the medical records: general and anthropometric information, symptoms, chest radiographs or CT films, serological indicators, and pulmonary function parameters. The chest radiographs or CT films were read at each site by two investigators; the findings were evaluated and staged according to the modified Scadding criteria.[10] Pulmonary function studies were

For peer review only - http://bmjopen.bmj.com/site/about/guidelines.xhtml

performed and percent predicted calculated,[11] airflow limitation was defined as an forced expiratory volume in one second/forced vital capacity ratio (FEV1/FVC ratio) below the 5th percentile of the predicted value; and the restrictive pattern was defined as forced vital capacity (FVC) below the predicted lower limit, with a normal FEV1/FVC ratio. The patients were followed up on chest HRCT at the outpatient clinic of Shanghai Pulmonary Hospital. The HRCT results were assessed by two independent radiologists. The deteriorating chest HRCT findings (an increase in extent compared with the previous HRCT results) were recorded in patients after their discharge from the hospital (figure 1). Written informed consent was obtained from all of the patients. This study was approved by the Ethics Committee of Shanghai Pulmonary Hospital. All aspects of the study were performed in accordance with relevant guidelines and regulations.

Statistical Analysis

Quantitative variables were expressed as the mean± standard deviation (SD) and qualitative variables were expressed as absolute numbers and percentages. The Kolmogorov–Smirnov test was used to analyze the distribution of variables. In the bivariate analysis, the t-test for independent variables was used to analyze normally distributed variables and the Mann-Whitney U-test was used to analyze non-normally distributed variables. Qualitative variables were compared using the Chi-squared test. A logistic regression model was used to determine the factors that were independently associated with deteriorating chest HRCT findings. The variables that presented statistically significant differences ($p<0.05$) in the bivariate analysis and were of clinical interest were included as the independent variables in the model. The odds ratio (OR) and 95% confidence interval were calculated for the independent variables. Statistical analysis was performed using SPSS 22.0 package software with a value of $p<0.05$ considered statistically significant, and graphs were drawn using GraphPad Prism 5 Software.

Results

A total of 636 biopsy proven sarcoidosis patients (378 isolated pulmonary sarcoidosis and 258 pulmonary sarcoidosis with extrapulmonary involvement) were included in the study. Among these 636 patients, 298 responded fully to the follow-up study on HRCT (182 isolated pulmonary sarcoidosis and 116 pulmonary sarcoidosis with extrapulmonary involvement), the median follow-up time after discharge was 30 months (ranging from 24 to 42 months). Thirty-nine patients had deteriorating chest HRCT findings after their discharge from the hospital.

The organ involvement and baseline characteristics of the subjects were shown in tables 1 and 2; the serological and pulmonary function parameters were shown in tables 3 and 4, respectively. Extrapulmonary localizations were detected mostly in extrathoracic lymph nodes (n=147) and skin (n=86). There were statistical differences in body mass index (BMI), fatigue, Scadding stage distribution, serum calcium (SC), SACE and globulin (GLB) levels. The airflow limitation was observed in 42.3% of patients with isolated pulmonary sarcoidosis patients and 42.2% of patients with pulmonary sarcoidosis and extrapulmonary involvement; there was no statistical significance between these two groups (p=0.984). Similar result was seen in volume restriction between these two groups (7.1% vs. 4.7%, p=0.198).

The differential characteristics of patients with or without deteriorating chest HRCT findings were shown in figure 2. Pulmonary sarcoidosis patients with extrapulmonary involvement had more deteriorating HRCT findings than patients with isolated pulmonary sarcoidosis (21 vs. 18, p=0.04). Even though corticosteroid therapy, FVC% predicted and FEV1/FVC ratio were not statistically significant, to

For peer review only

investigate whether these factors had influence on prognosis of HRCT, we set these parameters as independent variables in the logistic regression model.

The OR value and 95% confidence intervals of variables related to deteriorating HRCT findings in all patients were illustrated in Table 5. However, the extrapulmonary involvement, corticosteroid therapy, FVC% predicted, and FEV1/FVC ratio were irrelevant to the risk of deteriorating HRCT findings in these patients.

Discussion

The present study aimed to retrospectively compare the clinical characteristics of Chinese pulmonary sarcoidosis patients with and without extrapulmonary involvement as well as their prognosis on HRCT. So far, several studies conducted by different countries have been undertaken to investigate extrapulmonary sarcoidosis except for China. Since clinical features of sarcoidosis have varied among various populations around the world, our study would provide valuable information and therefore, may help facilitate the prognosis and management for Chinese sarcoidosis patients.

The prevalence of extrapulmonary sarcoidosis involvement varies among populations. Based on our study, we found that 40.6% pulmonary sarcoidosis patients had extrapulmonary involvement, which was consistent with previous study in which 30–50% of patients showed extrapulmonary disease localisations.[7] The most common sites found in our study were extrathoracic lymph nodes and skin, which is similar to studies from Turkey,[12,13] but differs from other researches. ACCESS demonstrated that Caucasians more frequently had a sarcoidosis-related calcium metabolism disorder;[14] Finnish and Japanese patients were found to have higher rates of cardiac and eye involvement;[15] Puerto Ricans were common with Lupus pernio skin lesions; and Europeans had more erythema nodosum lesions.[1] Extrapulmonary sarcoidosis may be more frequent in females, illustrated by a health maintenance organization database,[16] as well as a study from Beijing Tongren Hospital (Beijing, China).[17] However, we did not find the difference between gender in this study.

The most common symptom of mediastinal–pulmonary manifestations of sarcoidosis is persistent cough. Dyspnea is rare in the early stages, but more frequent later on.[3] Another symptom is fatigue that occurs in up to 70% of patients, which can be measured and monitored with a validated fatigue assessment scale,[18] which could predict quality of life.[19] Previous research found that pulmonary plus extrapulmonary sarcoidosis had higher Fatigue Scale total score and lower Baseline Dyspnea Index than isolated pulmonary sarcoidosis, demonstrated differences in the severity of symptoms.[20] In our study, a portion of patients have no symptoms, so we analyzed the proportion of various complaint, and found that extrapulmonary involvement patients had higher proportion of fatigue than isolated pulmonary sarcoidosis patients (16.6% vs. 8.3%, p=0.013). Neither cough nor dyspnea was found different between the two groups in our study.

Of note, in this study we found pulmonary sarcoidosis patients with extrapulmonary involvement had elevated serum ACE (SACE) level than patients with isolated pulmonary sarcoidosis (69.7±38.7 vs. 79.0±46.9 IU/L, p=0.016). Angiotensin convertase enzyme (ACE) is a caboxypeptidase enzyme that converts angiotensin I into angiotensin II,[21] which is produced in the epitheliod cell of the sarcoid granuloma.[22] SACE level, especially the amount of ACE activity, has been proposed as a biomarker that reflects the burden of granulomas.[23,24] Previous research also revealed that SACE level had correlated with extraparenchymal organs involvement and overall disease activity.[25,26] SACE is related to the various

For peer review only - http://bmjopen.bmj.com/site/about/guidelines.xhtml

polymorphisms of the ACE gene, so different races may have significant differences in their SACE levels.[27] Another significant biomarker in sarcoidosis is soluble interleukin 2 receptor (sIL-2R). A previous study showed that sIL-2R was higher in patients with extraparenchymal sarcoidosis than those with isolated pulmonary sarcoidosis.[28] However, in our study, there was no difference in sIL-2R concentration (1151±842 vs. 1163±798 U/ml in patients with and without extrapulmonary involvement, respectively, $p=0.32$). Further studies may be necessary to demonstrate the connection between serum biomarkers and differences in races and phenotypes.

Using chest radiograph or CT to view the internal structure is the most common imaging biomarker for evaluating sarcoidosis. The sarcoidosis granulomas can be detected with plain radiographic or computed tomography radiographic techniques.[29] In our study, isolated pulmonary sarcoidosis patients had higher proportion in stage I (n=128, $p<0.05$), while sarcoidosis patients with extrapulmonary involvement had higher proportion in stage II (n=180, $p<0.05$). Even though Scadding criteria provided useful prognostic information, these stages do not necessarily progress or regress from one to another.[30] CT is useful in assessing disease extent;[31] HRCT may also provide prognostic information.[32,33] However, CJ. Zappala et al[34] found that change in radiographic extent was more applicable to routine monitoring in sarcoidosis than change in radiographic stage. So we used deteriorating chest HRCT findings (an increase in extent compared with the previous HRCT results) as prognosis of sarcoidosis. FVC is the most common end point in pulmonary sarcoidosis trials;[35] significant airway obstruction is also associated with sarcoidosis via different mechanisms.[27] Corticosteroids are considered the drug for the treatment of almost all forms of sarcoidosis.[36] Even though these three factors were not statistically significant between patients with and without deteriorating HRCT findings, we still considered and included them as independent variables in the logistic regression model. However, we did not find relevant risk factors of deteriorating HRCT findings in these patients.

There are several limitations to this study. Firstly, there is a risk of selection bias due to the retrospective design. So we conducted a cross-sectional and observational study which might reduce the risk of selection bias. Secondly, we conducted this study more from a pulmonary perspective, since sarcoidosis is a multisystem disease, a more comprehensive study could be performed to investigate other parameters related to sarcoidosis. Thirdly, to date, no reliable prognostic biomarkers have been identified. Based on the previous research,[34] we used deteriorating chest HRCT findings as a prognostic factor of sarcoidosis. Further studies are needed to demonstrate potential biomarkers that would predict the prognosis.

In summary, our results suggest that extrapulmonary involvement is common in Chinese sarcoidosis patients, with the most common sites being extrathoracic lymph nodes and skin. Sarcoidosis patients with extrapulmonary involvement have more complaint of fatigue, higher SACE levels and more deteriorating HRCT findings than those with isolated pulmonary sarcoidosis, to which clinicians should pay attention.

Data Sharing Statement: No additional data available.

Contributorship Statement: Wei-Jun Cao has full access to all of the data in the study and takes responsibility for the integrity of the data and the accuracy of the data analysis. Cheng-Wei Li, Ru-Jia Tao and Dan-Feng Zou are joint first authors. Wei-Jun Cao and Xin Xu contributed to the study concept and design; Ru-Jia Tao and Man-Hui Li contributed to data collection and interpretation; Cheng-Wei Li and Dan-Feng Zou performed the statistical analyses; finally all authors discussed data results and

For peer review only

approved this submission.
Financial support: This research was funded by the National Key Research and Development Plan of Precision Medicine Project (Grant No. 2016YFC0905700).
Role of the Sponsors: The funding source had no role in the design and conduct of the study; collection, management, analysis, and interpretation of the data; preparation, review, or approval of the manuscript; and decision to submit the manuscript for publication.
Conflict of Interest: The authors have declared that no conflict of interest exists.

Reference

1. Hunninghake GW, Costabel U, Ando M, et al. ATS/ERS/WASOG statement on sarcoidosis. American Thoracic Society/European Respiratory Society/World Association of Sarcoidosis and other Granulomatous Disorders. *Sarcoidosis Vasc Diffuse Lung Dis* 1999; 16:149-73.
2. Baughman RP, Lower EE, du Bois RM. Sarcoidosis. *Lancet* 2003; 361:1111-8.
3. Valeyre D, Prasse A, Nunes H, et al. Sarcoidosis. *Lancet* 2014; 383:1155-67.
4. Bauer MP, Brouwer PA, Smit VT, et al. The challenges of extrapulmonary presentations of sarcoidosis: A case report and review of diagnostic strategies. *Eur J Intern Med* 2007; 18:152-4.
5. Rao DA, Dellaripa PF. Extrapulmonary manifestations of sarcoidosis. *Rheum Dis Clin North Am* 2013; 39:277-97.
6. Baughman R, Teirstein A, Ma, Rossman M, et al. Clinical characteristics of patients in a case control study of sarcoidosis. *American Journal of Respiratory & Critical Care Medicine* 2001; 164:1885-9.
7. Judson MA. Extrapulmonary sarcoidosis. *Seminars in Respiratory Infections* 2007; 28:83-101.
8 .Judson MA, Baughman RP, Teirstein AS, et al. Defining organ involvement in sarcoidosis: the ACCESS proposed instrument. ACCESS Research Group. A Case Control Etiologic Study of Sarcoidosis. *Sarcoidosis Vasc Diffuse Lung Dis* 1999; 16:75-86.
9. Judson MA, Costabel U, Drent M, et al. The WASOG Sarcoidosis Organ Assessment Instrument: An update of a previous clinical tool. *Sarcoidosis Vasc Diffuse Lung Dis* 2014; 31:19-27.
10. Scadding JG. Prognosis of intrathoracic sarcoidosis in England. A review of 136 cases after five years' observation. *Br Med J* 1961; 2:1165-72.
11. Hankinson JL, Odencrantz JR, Fedan KB. Spirometric reference values from a sample of the general U.S. population. *Am J Respir Crit Care Med* 1999; 159:179-87.
12. Okumus G, Musellim B, Cetinkaya E, et al. Extrapulmonary involvement in patients with sarcoidosis in Turkey. *Respirology* 2011; 16:446-50.
13. Zurkova M, Kolek V, Tomankova T, et al. Extrapulmonary involvement in patients with sarcoidosis and comparison of routine laboratory and clinical data to pulmonary involvement. *Biomed Pap Med Fac Univ Palacky Olomouc Czech Repub* 2014; 158:613-20.
14. Baughman RP, Teirstein AS, Judson MA, et al. Clinical characteristics of patients in a case control study of sarcoidosis. *Am J Respir Crit Care Med* 2001; 164:1885-9.
15. Pietinalho A, Ohmichi M, Hirasawa M, et al. Familial sarcoidosis in Finland and Hokkaido, Japan--a comparative study. *Respir Med* 1999; 93:408-12.
16. Rybicki BA, Major M, Popovich J, Jr., et al. Racial differences in sarcoidosis incidence: a 5-year study in a health maintenance organization. *Am J Epidemiol* 1997; 145:234-41.
17. Jin JM, Sun YC, Zhuo J, et al. A comparative clinical analysis of thoracic sarcoidosis and multi-organ sarcoidosis. *Zhonghua Jie He He Hu Xi Za Zhi* 2011; 34:914-8.
18. De Vries J, Michielsen H, Van Heck GL, et al. Measuring fatigue in sarcoidosis: the Fatigue Assessment Scale (FAS). *Br J Health Psychol* 2004; 9:279-91.
19. Michielsen HJ, Peros-Golubicic T, Drent M, et al. Relationship between symptoms and quality of life in a sarcoidosis population. *Respiration* 2007; 74:401-5.
20. Gvozdenovic BS, Mihailovic-Vucinic V, Ilic-Dudvarski A, et al. Differences in symptom severity and health status impairment between patients with pulmonary and pulmonary plus extrapulmonary sarcoidosis. *Respir Med* 2008; 102:1636-42.
21. Ryan JW, Smith U, Niemeyer RS. Angiotensin I: metabolism by plasma membrane of lung. *Science* 1972; 176:64-6.
22. Sheffield EA. Pathology of sarcoidosis. *Clin Chest Med* 1997; 18:741-54.
23. Lynch JP, Kazerooni EA, Gay SE. Pulmonary sarcoidosis. *Clin Chest Med* 1997; 18:755-85.

For peer review only

24. Gilbert S, Steinbrech DS, Landas SK, et al. Amounts of angiotensin-converting enzyme mRNA reflect the burden of granulomas in granulomatous lung disease. *Am Rev Respir Dis* 1993; 148:483-6.
25. Klech H, Kohn H, Kummer F, et al. Assessment of activity in Sarcoidosis. Sensitivity and specificity of 67Gallium scintigraphy, serum ACE levels, chest roentgenography, and blood lymphocyte subpopulations. *Chest* 1982; 82:732-8.
26. Selroos OB. Value of biochemical markers in serum for determination of disease activity in sarcoidosis. *Sarcoidosis* 1984; 1:45-9.
27. Chopra A, Kalkanis A, Judson MA. Biomarkers in sarcoidosis. *Expert Rev Clin Immunol* 2016; 12:1191-208.
28. Grutters JC, Fellrath JM, Mulder L, et al. Serum soluble interleukin-2 receptor measurement in patients with sarcoidosis: a clinical evaluation. *Chest* 2003; 124:186-95.
29. Nakatsu M, Hatabu H, Morikawa K, et al. Large coalescent parenchymal nodules in pulmonary sarcoidosis: "sarcoid galaxy" sign. *AJR Am J Roentgenol* 2002; 178:1389-1393.
30. Keijsers RG, Veltkamp M, Grutters JC. Chest Imaging. *Clin Chest Med* 2015; 36:603-19.
31. Greco FG, Spagnolo P, Muri M, et al. The value of chest radiograph and computed tomography in pulmonary sarcoidosis. *Sarcoidosis Vasc Diffuse Lung Dis* 2014; 31:108-16.
32. Akira M, Kozuka T, Inoue Y, et al. Long-term follow-up CT scan evaluation in patients with pulmonary sarcoidosis. *Chest* 2005; 127:185-91.
33. Keir G, Wells AU. Assessing pulmonary disease and response to therapy: which test? *Semin Respir Crit Care Med* 2010; 31:409-18.
34. Zappala CJ, Desai SR, Copley SJ, et al. Optimal scoring of serial change on chest radiography in sarcoidosis. Sarcoidosis Vasc Diffuse Lung Dis. 2011; 28:130-8.
35. Baughman RP, Drent M, Culver DA, et al. Endpoints for clinical trials of sarcoidosis. Sarcoidosis Vasc Diffuse Lung Dis. 2012; 29:90-8.
36. Judson MA. Corticosteroids in Sarcoidosis. *Rheum Dis Clin North Am* 2016; 42:119-35, ix.

For peer review only

Table 1. Organ involvement in sarcoidosis patients

Organ	No. (%)
Isolated pulmonary involvement	378 (59.4)
Pulmonary sarcoidosis with extrapulmonary involvement	258 (40.6)
Extrathoracic lymph node	147 (23.1)
Skin	86 (13.5)
Eyes	8 (1.3)
Bone/joints	8 (1.3)
Liver	4 (0.6)
Nose	4 (0.6)
Parotid/salivary	3 (0.5)
Spleen	2 (0.3)
Renal	2 (0.3)
Cardiac	1 (0.2)
Muscles	1 (0.2)

Total numbers add up to greater than the total number of patients because 8 patients had more than one organ involvement.

For peer review only

Table 2. Baseline and clinical characteristics of subjects with pulmonary sarcoidosis, with and without extrapulmonary involvement

Parameter	Isolated pulmonary sarcoidosis	Pulmonary sarcoidosis with extrapulmonary involvement	p-value
Subjects, No. (%)	378 (59.4)	258 (40.6)	—
Sex, M/F, No.	260/118	174/84	0.721
Age, y	48.8±10.7	48.2±11.5	0.613
BMI, kg/m^2	23.9±3.3	23.4±3.3	0.033
Smoking history, No. (%)	59 (15.6)	29 (11.2)	0.114
Occupational exposure, No. (%)	33 (8.7)	18 (7.0)	0.424
Symptoms, No. (%)	277 (73.3)	181 (70.2)	0.467
Cough, No. (%)	231 (83.4)	142 (78.5)	0.127
Chest congestion, No. (%)	80 (28.9)	50 (27.6)	0.548
Dyspnea, No. (%)	50 (18.1)	36 (19.9)	0.793
Chest pain, No. (%)	39 (14.1)	19 (10.5)	0.204
Fatigue, No. (%)	23 (8.3%)	30 (16.6%)	0.013
Fever, No. (%)	18 (6.5)	10 (5.5)	0.593
Scadding stage			
Stage 1, No. (%)	128（33.9）	67（25.9）	0.034
Stage 2, No. (%)	232（61.4）	180（69.8）	0.0296
Stage 3, No. (%)	18（4.7）	11（4.3）	0.7674

Data are presented as mean±SD or %. BMI: body mass index.

For peer review only

Table 3. Serological indicators of subjects with pulmonary sarcoidosis, with and without extrapulmonary involvement

Parameter	Isolated pulmonary sarcoidosis	Pulmonary sarcoidosis with extrapulmonary involvement	p-value
CRP, mg/L	4.0±4.2	3.8±3.0	0.547
TB-AB, No. %	22 (5.8)	20 (7.8)	0.335
SIL-2R, U/ml	1151±842	1163±798	0.32
UC, mmol/24h	6.09±2.94	6.08±2.65	0.117
SC, mmol/L	2.39±0.13	2.42±0.15	0.022
SACE, IU/L	69.7±38.7	79.0±46.9	0.016
ALP, IU/L	86.4±33.4	84.7±33.4	0.259
ALB, g/L	40.16±4.02	40.81±3.86	0.8
GLB, g/L	27.96±5.34	30.14±5.67	0.013
A/G	1.47±0.28	1.42±0.27	0.064
UA,umol/L	309.4±85.4	308.6±99.3	0.728
Seroglobulin β	11.23±1.71	11.30±1.43	0.278
Seroglobulin γ	17.72±3.93	18.40±4.13	0.084
WBC,10^9/L	6.2±2.1	5.7±2.0	0.377
ESR,mm/H	15.8±10.7	15.8±9.3	0.288

Data are presented as mean±SD or %. CRP: C-reactive protein; TB-AB: tuberculosis antibody; SIL-2R: soluble interleukin 2 receptor; UC: urinary calcium; SC: serum calcium; SACE: serum angiotensin converting enzyme; ALP: alkaline phosphatase; ALB: albumin; GLB: globulin; A/G: albumin/globulin ratio; UA: blood uric acid; WBC: white blood count; ESR: erythrocyte sedimentation rate.

For peer review only

Table 4. Pulmonary function parameters of subjects with pulmonary sarcoidosis, with and without extrapulmonary involvement

Parameter	Isolated pulmonary sarcoidosis	Pulmonary sarcoidosis with extrapulmonary involvement	p-value
VC, L	3.04±0.75	3.09±0.82	0.327
FVC, L	3.03±0.75	3.07±0.81	0.309
FVC % pred	97.5 ± 15.6	96.8 ± 17.2	0.305
FEV1, L	3.03±0.75	3.07±0.81	0.21
FEV1 % pred	88.61 ± 15.08	88.89 ± 16.30	0.452
FEV1/FVC	78.62±7.74	78.94±8.52	0.072
RV, L	1.84±0.43	1.83±0.55	0.317
TLC, L	4.86±0.93	4.89±1.03	0.972
RV/TLC	38.51 ± 7.66	37.73 ± 9.16	0.137
DLCO, ml/min/mmHg	21.1 ± 4.8	20.6 ± 5.2	0.487
Airflow limitation, No. (%)	160 (42.3)	109 (42.2)	0.984
Volume restriction, No. (%)	27 (7.1)	12 (4.7)	0.198

Data are presented as mean±SD or %. VC: vital capacity; FVC: forced vital capacity; FEV1: forced expiratory volume in 1 s; RV: residual volume; TLC: total lung capacity; DLCO: diffusion capacity for carbon monoxide of lung.

For peer review only

Table 5. Factors associated with deteriorating HRCT findings in all subjects according to the logistic regression analysis

Parameter	OR (95% CI)	p-value
Extrapulmonary involvement	0.516（0.232-1.147）	0.104
Corticosteroid therapy	0.941（0.42-2.11）	0.884
FVC% pre	0.998（0.978-1.108）	0.843
FEV1/FVC ratio	0.998（0.954-1.043）	0.928

FVC: forced vital capacity; FEV1: forced expiratory volume in 1 s.

Legend of figures

Figure 1. HRCT findings in sarcoidosis patients. (a) Shows pulmonary micronodules at the time of initial hospitalization; (b) with pulmonary involvement during follow-up. (c) Shows enlarged mediastinal lymph node at the time of initial hospitalization; (d) The mediastinal lymph node became larger during follow-up.

Figure 2. (a) Pulmonary sarcoidosis with or without extrapulmonary involvement; (b) with or without corticosteroid therapy; (c) forced vital capacity (FVC) % predicted; (d) forced expiratory volume in 1 s (FEV1) / FVC ratio of patients who had deteriorating chest radiograph findings and patients who did not have deteriorating chest radiograph findings.

For peer review only

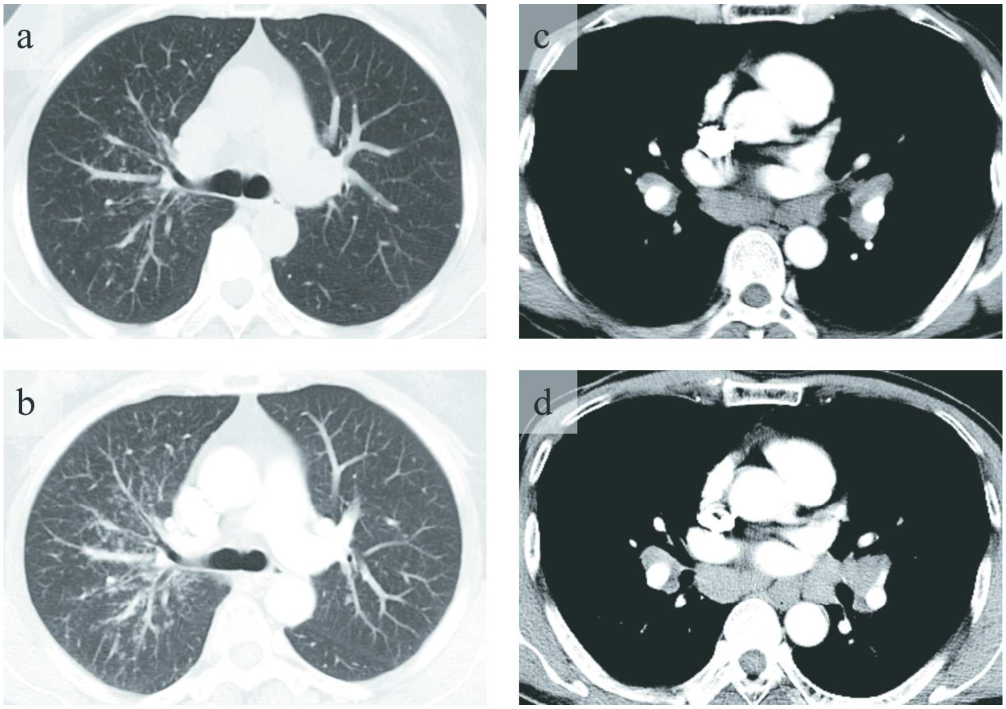

Figure 1. HRCT findings in sarcoidosis patients. (a) Shows pulmonary micronodules at the time of initial hospitalization; (b) with pulmonary involvement during follow-up. (c) Shows enlarged mediastinal lymph node at the time of initial hospitalization; (d) The mediastinal lymph node became larger during follow-up.

268x188mm (300 x 300 DPI)

review only

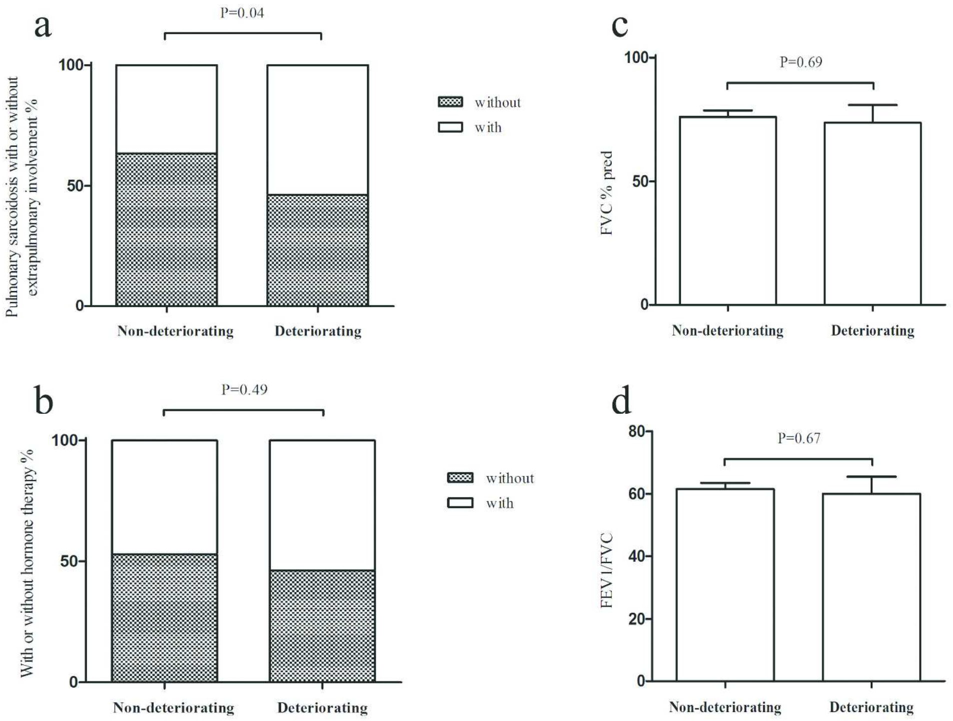

Figure 2. (a) Pulmonary sarcoidosis with or without extrapulmonary involvement; (b) with or without corticosteroid therapy; (c) forced vital capacity (FVC) % predicted; (d) forced expiratory volume in 1 s (FEV1) / FVC ratio of patients who had deteriorating chest radiograph findings and patients who did not have deteriorating chest radiograph findings.

256x190mm (300 x 300 DPI)

STROBE Statement—Checklist of items that should be included in reports of ***cross-sectional studies***

	Item No	Recommendation
**Title and abstract**	1	(*a*) Indicate the study's design with a commonly used term in the title or the abstract P1
		(*b*) Provide in the abstract an informative and balanced summary of what was done and what was found P2
**Introduction**		
Background/rationale	2	Explain the scientific background and rationale for the investigation being reported P4
Objectives	3	State specific objectives, including any prespecified hypotheses P4
**Methods**		
Study design	4	Present key elements of study design early in the paper P4
Setting	5	Describe the setting, locations, and relevant dates, including periods of recruitment, exposure, follow-up, and data collection P4
Participants	6	(*a*) Give the eligibility criteria, and the sources and methods of selection of participants P4
Variables	7	Clearly define all outcomes, exposures, predictors, potential confounders, and effect modifiers. Give diagnostic criteria, if applicable P4
Data sources/ measurement	8*	For each variable of interest, give sources of data and details of methods of assessment (measurement). Describe comparability of assessment methods if there is more than one group P4 P5
Bias	9	Describe any efforts to address potential sources of bias P7
Study size	10	Explain how the study size was arrived at P4
Quantitative variables	11	Explain how quantitative variables were handled in the analyses. If applicable, describe which groupings were chosen and why P4 P5
Statistical methods	12	(*a*) Describe all statistical methods, including those used to control for confounding P5
		(*b*) Describe any methods used to examine subgroups and interactions P5
		(*c*) Explain how missing data were addressed NONE
		(*d*) If applicable, describe analytical methods taking account of sampling strategy P5
		(*e*) Describe any sensitivity analyses P5
**Results**		
Participants	13*	(a) Report numbers of individuals at each stage of study—eg numbers potentially eligible, examined for eligibility, confirmed eligible, included in the study, completing follow-up, and analysed P5 P6
		(b) Give reasons for non-participation at each stage NONE
		(c) Consider use of a flow diagram NONE
Descriptive data	14*	(a) Give characteristics of study participants (eg demographic, clinical, social) and information on exposures and potential confounders P5
		(b) Indicate number of participants with missing data for each variable of interest P5 P6
Outcome data	15*	Report numbers of outcome events or summary measures P5 P6
Main results	16	(*a*) Give unadjusted estimates and, if applicable, confounder-adjusted estimates and their precision (eg, 95% confidence interval). Make clear which confounders were adjusted for and why they were included P6
		(*b*) Report category boundaries when continuous variables were categorized P5 P6

For peer review only

		(*c*) If relevant, consider translating estimates of relative risk into absolute risk for a meaningful time period P6
Other analyses	17	Report other analyses done—eg analyses of subgroups and interactions, and sensitivity analyses P5 P6
**Discussion**		
Key results	18	Summarise key results with reference to study objectives P6 P7
Limitations	19	Discuss limitations of the study, taking into account sources of potential bias or imprecision. Discuss both direction and magnitude of any potential bias P7
Interpretation	20	Give a cautious overall interpretation of results considering objectives, limitations, multiplicity of analyses, results from similar studies, and other relevant evidence P6 P7
Generalisability	21	Discuss the generalisability (external validity) of the study results P6 P7
**Other information**		
Funding	22	Give the source of funding and the role of the funders for the present study and, if applicable, for the original study on which the present article is based P8

*Give information separately for exposed and unexposed groups.

For peer review only

BMJ Open

Pulmonary sarcoidosis with and without extrapulmonary involvement : A cross-sectional and observational study

Journal:	*BMJ Open*
Manuscript ID	bmjopen-2017-018865.R1
Article Type:	Research
Date Submitted by the Author:	28-Dec-2017
Complete List of Authors:	li, chengwei; Shanghai Pulmonary Hospital, Tongji University School of Medicine, Department of Respiratory and Critical Care Medicine<br>tao, rujia; Shanghai Pulmonary Hospital, Tongji University School of Medicine, Department of Respiratory and Critical Care Medicine<br>zou, danfeng; Shanghai Pulmonary Hospital, Tongji University School of Medicine, Clinic and Research Center of Tuberculosis, Shanghai Key Lab of Tuberculosis<br>li, manhui; Shanghai Pulmonary Hospital, Tongji University School of Medicine, Department of Respiratory and Critical Care Medicine<br>xu, xin; Shanghai Pulmonary Hospital, Tongji University School of Medicine, Department of Respiratory and Critical Care Medicine<br>cao, weijun; Tongji University Affiliated Shanghai Pulmonary Hospital, Department of Respiratory Medicine
**Primary Subject Heading**:	Respiratory medicine
Secondary Subject Heading:	Respiratory medicine, Radiology and imaging
Keywords:	sarcoidosis, extrapulmonary involvement, fatigue, prognosis

SCHOLARONE™
Manuscripts

Pulmonary sarcoidosis with and without extrapulmonary involvement：A cross-sectional and observational study

Cheng-Wei Li[a#], Ru-Jia Tao[b#], Dan-Feng Zou[c#], Man-Hui Li[b], Xin Xu[b], Wei-Jun Cao[b]

Institution:
a: Department of Respiratory and Critical Care Medicine, Shanghai Pulmonary Hospital, Soochow University, Suzhou 215006, China
b: Department of Respiratory and Critical Care Medicine, Shanghai Pulmonary Hospital, Tongji University School of Medicine, Shanghai 200433, China
c: Clinic and Research Center of Tuberculosis, Shanghai Key Lab of Tuberculosis, Shanghai Pulmonary Hospital, Tongji University School of Medicine, Shanghai 200433, China
#: These authors contributed equally.

Corresponding author: Wei-Jun Cao, Department of Respiratory and Critical Care Medicine, Shanghai Pulmonary Hospital, Tongji University School of Medicine, No. 507 Zhengmin Road, Shanghai, 200433, China. E-mail: weijuncao@126.com

Word count: 2,042 words.

For peer review only

ABSTRACT

Objectives: Sarcoidosis is a multisystem disease characterized by the formation of granulomas within various organs, mainly the lungs. Several studies from different countries have been undertaken to investigate sarcoidosis with extrapulmonary involvement but from China. The objective of this study is to investigate a comparative clinical analysis in pulmonary sarcoidosis patients with and without extrapulmonary involvement from China.

Methods: Data from inpatients diagnosed with sarcoidosis at Shanghai Pulmonary Hospital (Shanghai, China) between January 2009 and December 2014 were retrospectively collected and analyzed. Six hundred and thirty-six biopsy proven sarcoidosis patients were included in the study, including 378 isolated pulmonary sarcoidosis and 258 pulmonary sarcoidosis plus extrapulmonary involvement.

Results: Two hundred and fifty eight (40.6%) pulmonary sarcoidosis patients had extrapulmonary involvement. Extrapulmonary localizations were detected mostly in extrathoracic lymph nodes (n=147) and skin (n=86). Statistically significant differences were demonstrated between patients with pulmonary plus extrapulmonary involvement and patients with isolated pulmonary sarcoidosis for fatigue (16.6% vs. 8.3%, $p<0.05$), serum angiotensin-converting enzyme (SACE) levels (79.0±46.9 IU/L vs. 69.7±38.7 IU/L, $p<0.05$), and high-resolution computed tomography (HRCT) findings (53.8% vs. 46.2%, $p<0.05$).

Conclusions:Extrapulmonary involvement was common in pulmonary sarcoidosis patients, with the most common sites being extrathoracic lymph nodes and skin. Sarcoidosis patients with extrapulmonary involvement were more symptomatic (fatigue), had higher SACE levels and more deteriorating HRCT findings, to which clinicians should pay attention.

Keywords: sarcoidosis; extrapulmonary involvement; fatigue; prognosis

For peer review only

Article summary

Strengths and limitations of this study

1. The present study provides a large number of pulmonary sarcoidosis patients with and without extrapulmonary involvement from China.
2. The sarcoidosis patients had a long term followe-up (24-42 months) on chest HRCT.
3. There is a risk of selection bias due to the retrospective design.

For peer review only

INTRODUCTION

Sarcoidosis is a granulomatous disease of unknown etilogy. It is characterized by the formation of immune granulomas in many involved organs.[1,2] Sarcoidosis is a worldwide disease, with its prevalence ranging from 4.7 to 64 in 100 000, and its incidence from 1.0 to 35.5 in 100 000 per year.[3] However, few epidemiological data in China is not available.

Sarcoidosis is a multisystem disease. Although the lungs are most commonly involved, pulmonary sarcoidosis is often accompanied by the involvement of extrapulmonary organs,[1,4] such as the skin, eyes, and heart that are significantly affected.[5] A Case Control Etiologic Study of Sarcoidosis (ACCESS) showed that among 736 patients with sarcoidosis, 95% had pulmonary involvement, and 368 out of these 736 patients (50%) had concomitant extrapulmonary involvement.[6] Extrapulmonary involvement can be the major presentation of the disease and sometimes may be life-threatening.[5,7] Moreover, the assessment of pulmonary and extrapulmonary organ involvement is important for clinical treatment decisions.[8]

It is well known that the features of sarcoidosis have varied widely among various populations around the world. To date, several studies from different countries have been undertaken to investigate sarcoidosis with extrapulmonary involvement. However, there is limited information in Chinese sarcoidosis patients. Therefore, the purpose of this study is to compare the clinical characteristics and prognosis of pulmonary sarcoidosis patients with and without extrapulmonary involvement from China.

METHODS

Data were retrospectively collected from inpatients diagnosed with sarcoidosis at the Shanghai Pulmonary Hospital (Shanghai, China) between January 2009 and December 2014. Patients who had not received a chest radiograph or chest high resolution computed tomography (HRCT) scan examination or who had not received tissue biopsy were excluded. All aspects of the study were performed in accordance with relevant guidelines and regulations.

Diagnosis of sarcoidosis is based on standardized criteria,[1] which detailed the features of sarcoidosis, including clinical and radiological presentation, and pathological findings (noncaseating epithelioid granulomas), as well as evidence of no alternative diseases (infections, particularly tuberculosis; occupationally induced, environmentally induced, and drug-induced granulomatosis; common variable immune deficiency; Blau's syndrome; sarcoid-like reactions in cancers and lymphomas, and other idiopathic granulomatosis).[3]

Organ involvement was determined and classified in each patient in accordance with criteria proposed in the ACCESS formulation,[9] based upon clinical assessment and widely available tests, including the evolution of electrocardiography, positron emission tomography (PET) scans as well as magnetic resonance imaging (MRI) that can improve the detection of disease. According to the organs affected by sarcoidosis, patients were divided into two groups: isolated pulmonary sarcoidosis and pulmonary sarcoidosis with extrapulmonary involvement.

The following variables were obtained from the medical records: general and anthropometric information, symptoms, chest radiographs or CT films, serological indicators, and pulmonary function parameters. The chest radiographs or CT films were read at each site by two investigators; the findings were evaluated and staged according to the modified Scadding criteria.[10] Pulmonary function studies were performed and percent predicted calculated,[11] airflow limitation was defined as an forced expiratory volume in one second/forced vital capacity ratio (FEV1/FVC ratio)

For peer review only

below the 5th percentile of the predicted value; and the restrictive pattern was defined as forced vital capacity (FVC) below the predicted lower limit, with a normal FEV1/FVC ratio. The patients were followed up on chest HRCT at the outpatient clinic of Shanghai Pulmonary Hospital. The HRCT results were assessed by two independent radiologists. The deteriorating chest HRCT findings (an increase in extent compared with the previous HRCT results) were recorded in patients after their discharge from the hospital (figure 1). Written informed consent was obtained from all of the patients. This study was approved by the Ethics Committee of Shanghai Pulmonary Hospital. All aspects of the study were performed in accordance with relevant guidelines and regulations.

Statistical Analysis

Quantitative variables were expressed as the mean± standard deviation (SD) and qualitative variables were expressed as absolute numbers and percentages. The Kolmogorov–Smirnov test was used to analyze the distribution of variables. In the bivariate analysis, the t-test for independent variables was used to analyze normally distributed variables and the Mann-Whitney U-test was used to analyze non-normally distributed variables. Qualitative variables were compared using the Chi-squared test. A logistic regression model was used to determine the factors that were independently associated with deteriorating chest HRCT findings. The variables that presented statistically significant differences ($p<0.05$) in the bivariate analysis and were of clinical interest were included as the independent variables in the model. The odds ratio (OR) and 95% confidence interval were calculated for the independent variables. Statistical analysis was performed using SPSS 22.0 package software with a value of $p <0.05$ considered statistically significant, and graphs were drawn using GraphPad Prism 5 Software.

Results

A total of 636 biopsy proven sarcoidosis patients (378 isolated pulmonary sarcoidosis and 258 pulmonary sarcoidosis with extrapulmonary involvement) were included in the study. Among these 636 patients, 298 responded fully to the follow-up study on HRCT (182 isolated pulmonary sarcoidosis and 116 pulmonary sarcoidosis with extrapulmonary involvement), the median follow-up time after discharge was 30 months (ranging from 24 to 42 months). Thirty-nine patients had deteriorating chest HRCT findings after their discharge from the hospital.

The organ involvement and baseline characteristics of the subjects were shown in tables 1 and 2; the serological and pulmonary function parameters were shown in tables 3 and 4, respectively. Extrapulmonary localizations were detected mostly in extrathoracic lymph nodes (n=147) and skin (n=86). There were statistical differences in body mass index (BMI), fatigue, Scadding stage distribution, serum calcium (SC), SACE and globulin (GLB) levels. The airflow limitation was observed in 42.3% of patients with isolated pulmonary sarcoidosis patients and 42.2% of patients with pulmonary sarcoidosis and extrapulmonary involvement; there was no statistical significance between these two groups ($p=0.984$). Similar result was seen in volume restriction between these two groups (7.1% vs. 4.7%, $p=0.198$).

The differential characteristics of patients with or without deteriorating chest HRCT findings were shown in figure 2. Pulmonary sarcoidosis patients with extrapulmonary involvement had more deteriorating HRCT findings than patients with isolated pulmonary sarcoidosis (21 vs. 18, $p=0.04$). Even though corticosteroid therapy, FVC% predicted and FEV1/FVC ratio were not statistically significant, to investigate whether these factors had influence on prognosis of HRCT, we set these parameters as independent variables in the logistic regression model.

For peer review only - http://bmjopen.bmj.com/site/about/guidelines.xhtml

The OR value and 95% confidence intervals of variables related to deteriorating HRCT findings in all patients were illustrated in Table 5. However, the extrapulmonary involvement, corticosteroid therapy, FVC% predicted, and FEV1/FVC ratio were irrelevant to the risk of deteriorating HRCT findings in these patients.

Discussion

The present study aimed to retrospectively compare the clinical characteristics of pulmonary sarcoidosis patients with and without extrapulmonary involvement as well as their prognosis on HRCT. So far, several studies conducted by different countries have been undertaken to investigate extrapulmonary sarcoidosis except for China. Since clinical features of sarcoidosis have varied among various populations around the world, our study would provide valuable information and therefore, may help facilitate the prognosis and management for Chinese sarcoidosis patients.

The prevalence of extrapulmonary sarcoidosis involvement varies among populations. Based on our study, we found that 40.6% pulmonary sarcoidosis patients had extrapulmonary involvement, which was consistent with previous study in which 30–50% of patients showed extrapulmonary disease localisations.[7] The most common sites found in our study were extrathoracic lymph nodes and skin, which is similar to studies from Turkey,[12,13] but differs from other researches. Skin lesions are divided into two categories: specific and nonspecific. In this study most of skin lesions are specific lesions that demonstrate granulomatous inflammation on biopsy. The rest are nonspecific lesions that show no granulomatous inflammation, such as erythema nodosum. ACCESS demonstrated that Caucasians more frequently had a sarcoidosis-related calcium metabolism disorder;[14] Finnish and Japanese patients were found to have higher rates of cardiac and eye involvement;[15] Puerto Ricans were common with Lupus pernio skin lesions; and Europeans had more erythema nodosum lesions.[1] Extrapulmonary sarcoidosis may be more frequent in females, illustrated by a health maintenance organization database,[16] as well as a study from Beijing Tongren Hospital (Beijing, China).[17] However, we did not find the difference between gender in this study.

The most common symptom of mediastinal–pulmonary manifestations of sarcoidosis is persistent cough. Dyspnea is rare in the early stages, but more frequent later on.[3] Another symptom is fatigue that occurs in up to 70% of patients, which can be measured and monitored with a validated fatigue assessment scale,[18] which could predict quality of life.[19] Previous research showed that patients with pulmonary and extrapulmonary sarcoidosis were more fatigued and more dyspneic than those with pulmonary involvement alone, which demonstrated differences in the severity of symptoms.[20] In our study, a portion of patients have no symptoms, so we analyzed the proportion of various complaint, and found that extrapulmonary involvement patients had higher proportion of fatigue than isolated pulmonary sarcoidosis patients (16.6% vs. 8.3%, p=0.013). Neither cough nor dyspnea was found different between the two groups in our study.

Of note, in this study we found pulmonary sarcoidosis patients with extrapulmonary involvement had elevated serum ACE (SACE) level than patients with isolated pulmonary sarcoidosis (79.0±46.9 vs. 69.7±38.7 IU/L, p=0.016). Angiotensin convertase enzyme (ACE) is a caboxypeptidase enzyme that converts angiotensin I into angiotensin II,[21] which is produced in the epitheliod cell of the sarcoid granuloma.[22] SACE level, especially the amount of ACE activity, has been proposed as a biomarker that reflects the burden of granulomas.[23,24] Previous research also revealed that SACE level had correlated with extraparenchymal organs

For peer review only - http://bmjopen.bmj.com/site/about/guidelines.xhtml

involvement and overall disease activity.[25,26] SACE is related to the various polymorphisms of the ACE gene, so different races may have significant differences in their SACE levels.[27] Another significant biomarker in sarcoidosis is soluble interleukin 2 receptor (sIL-2R). A previous study showed that sIL-2R was higher in patients with extraparenchymal sarcoidosis than those with isolated pulmonary sarcoidosis.[28] However, in our study, there was no difference in sIL-2R concentration (1151±842 vs. 1163±798 U/ml in patients with and without extrapulmonary involvement, respectively, p=0.32). Further studies may be necessary to demonstrate the connection between serum biomarkers and differences in races and phenotypes.

Using chest radiograph or CT to view the internal structure is the most common imaging biomarker for evaluating sarcoidosis. The sarcoidosis granulomas can be detected with plain radiographic or computed tomography radiographic techniques.[29] In our study, isolated pulmonary sarcoidosis patients had higher proportion in stage I (n=128, $p<0.05$), while sarcoidosis patients with extrapulmonary involvement had higher proportion in stage II (n=180, $p<0.05$). Even though Scadding criteria provided useful prognostic information, these stages do not necessarily progress or regress from one to another.[30] CT is useful in assessing disease extent;[31] HRCT may also provide prognostic information.[32,33] However, CJ. Zappala et al[34] found that change in radiographic extent was more applicable to routine monitoring in sarcoidosis than change in radiographic stage. So we used deteriorating chest HRCT findings (an increase in extent compared with the previous HRCT results) as prognosis of sarcoidosis. FVC is the most common end point in pulmonary sarcoidosis trials;[35] significant airway obstruction is also associated with sarcoidosis via different mechanisms.[27] Corticosteroids are considered the drug for the treatment of almost all forms of sarcoidosis.[36] Even though these three factors were not statistically significant between patients with and without deteriorating HRCT findings, we still considered and included them as independent variables in the logistic regression model. However, we did not find relevant risk factors of deteriorating HRCT findings in these patients.

There are several limitations to this study. Firstly, there is a risk of selection bias due to the retrospective design. So we conducted a cross-sectional and observational study which might reduce the risk of selection bias. Secondly, we conducted this study more from a pulmonary perspective, since sarcoidosis is a multisystem disease, a more comprehensive study could be performed to investigate other parameters related to sarcoidosis. Thirdly, to date, no reliable prognostic biomarkers have been identified. Based on the previous research,[34] we used deteriorating chest HRCT findings as a prognostic factor of sarcoidosis. Further studies are needed to demonstrate potential biomarkers that would predict the prognosis.

In summary, our results suggest that extrapulmonary involvement is common in pulmonary sarcoidosis patients, with the most common sites being extrathoracic lymph nodes and skin. Sarcoidosis patients with extrapulmonary involvement have more complaint of fatigue, higher SACE levels and more deteriorating HRCT findings than those with isolated pulmonary sarcoidosis, to which clinicians should pay attention.

Data Sharing Statement: No additional data available.

Contributorship Statement: Wei-Jun Cao has full access to all of the data in the study and takes responsibility for the integrity of the data and the accuracy of the data analysis. Cheng-Wei Li, Ru-Jia Tao and Dan-Feng Zou are joint first authors. Wei-Jun Cao and Xin Xu contributed to the study concept and design; Ru-Jia Tao and Man-

For peer review only

Hui Li contributed to data collection and interpretation; Cheng-Wei Li and Dan-Feng Zou performed the statistical analyses; finally all authors discussed data results and approved this submission.

Financial support: This research was funded by the National Key Research and Development Plan of Precision Medicine Project (Grant No. 2016YFC0905700).

Role of the Sponsors: The funding source had no role in the design and conduct of the study; collection, management, analysis, and interpretation of the data; preparation, review, or approval of the manuscript; and decision to submit the manuscript for publication.

Conflict of Interest: The authors have declared that no conflict of interest exists.

Reference

1. Hunninghake GW, Costabel U, Ando M, et al. ATS/ERS/WASOG statement on sarcoidosis. American Thoracic Society/European Respiratory Society/World Association of Sarcoidosis and other Granulomatous Disorders. *Sarcoidosis Vasc Diffuse Lung Dis* 1999; 16:149-73.
2. Baughman RP, Lower EE, du Bois RM. Sarcoidosis. *Lancet* 2003; 361:1111-8.
3. Valeyre D, Prasse A, Nunes H, et al. Sarcoidosis. *Lancet* 2014; 383:1155-67.
4. Bauer MP, Brouwer PA, Smit VT, et al. The challenges of extrapulmonary presentations of sarcoidosis: A case report and review of diagnostic strategies. *Eur J Intern Med* 2007; 18:152-4.
5. Rao DA, Dellaripa PF. Extrapulmonary manifestations of sarcoidosis. *Rheum Dis Clin North Am* 2013; 39:277-97.
6. Baughman R, Teirstein A, Ma, Rossman M, et al. Clinical characteristics of patients in a case control study of sarcoidosis. *American Journal of Respiratory & Critical Care Medicine* 2001; 164:1885-9.
7. Judson MA. Extrapulmonary sarcoidosis. *Seminars in Respiratory Infections* 2007; 28:83-101.
8 .Judson MA, Baughman RP, Teirstein AS, et al. Defining organ involvement in sarcoidosis: the ACCESS proposed instrument. ACCESS Research Group. A Case Control Etiologic Study of Sarcoidosis. *Sarcoidosis Vasc Diffuse Lung Dis* 1999; 16:75-86.
9. Judson MA, Costabel U, Drent M, et al. The WASOG Sarcoidosis Organ Assessment Instrument: An update of a previous clinical tool. *Sarcoidosis Vasc Diffuse Lung Dis* 2014; 31:19-27.
10. Scadding JG. Prognosis of intrathoracic sarcoidosis in England. A review of 136 cases after five years' observation. *Br Med J* 1961; 2:1165-72.
11. Hankinson JL, Odencrantz JR, Fedan KB. Spirometric reference values from a sample of the general U.S. population. *Am J Respir Crit Care Med* 1999; 159:179-87.
12. Okumus G, Musellim B, Cetinkaya E, et al. Extrapulmonary involvement in patients with sarcoidosis in Turkey. *Respirology* 2011; 16:446-50.
13. Zurkova M, Kolek V, Tomankova T, et al. Extrapulmonary involvement in patients with sarcoidosis and comparison of routine laboratory and clinical data to pulmonary involvement. *Biomed Pap Med Fac Univ Palacky Olomouc Czech Repub* 2014; 158:613-20.
14. Baughman RP, Teirstein AS, Judson MA, et al. Clinical characteristics of patients in a case control study of sarcoidosis. *Am J Respir Crit Care Med* 2001; 164:1885-9.
15. Pietinalho A, Ohmichi M, Hirasawa M, et al. Familial sarcoidosis in Finland and Hokkaido, Japan--a comparative study. *Respir Med* 1999; 93:408-12.
16. Rybicki BA, Major M, Popovich J, Jr., et al. Racial differences in sarcoidosis incidence: a 5-year study in a health maintenance organization. *Am J Epidemiol* 1997; 145:234-41.
17. Jin JM, Sun YC, Zhuo J, et al. A comparative clinical analysis of thoracic sarcoidosis and multi-organ sarcoidosis. *Zhonghua Jie He He Hu Xi Za Zhi* 2011; 34:914-8.
18. De Vries J, Michielsen H, Van Heck GL, et al. Measuring fatigue in sarcoidosis: the Fatigue Assessment Scale (FAS). *Br J Health Psychol* 2004; 9:279-91.
19. Michielsen HJ, Peros-Golubicic T, Drent M, et al. Relationship between symptoms and quality of life in a sarcoidosis population. *Respiration* 2007; 74:401-5.
20. Gvozdenovic BS, Mihailovic-Vucinic V, Ilic-Dudvarski A, et al. Differences in symptom severity and health status impairment between patients with pulmonary and pulmonary plus extrapulmonary sarcoidosis. *Respir Med* 2008; 102:1636-42.
21. Ryan JW, Smith U, Niemeyer RS. Angiotensin I: metabolism by plasma membrane of lung. *Science* 1972; 176:64-6.

For peer review only

22. Sheffield EA. Pathology of sarcoidosis. *Clin Chest Med* 1997; 18:741-54.
23. Lynch JP, Kazerooni EA, Gay SE. Pulmonary sarcoidosis. *Clin Chest Med* 1997; 18:755-85.
24. Gilbert S, Steinbrech DS, Landas SK, et al. Amounts of angiotensin-converting enzyme mRNA reflect the burden of granulomas in granulomatous lung disease. *Am Rev Respir Dis* 1993; 148:483-6.
25. Klech H, Kohn H, Kummer F, et al. Assessment of activity in Sarcoidosis. Sensitivity and specificity of 67Gallium scintigraphy, serum ACE levels, chest roentgenography, and blood lymphocyte subpopulations. *Chest* 1982; 82:732-8.
26. Selroos OB. Value of biochemical markers in serum for determination of disease activity in sarcoidosis. *Sarcoidosis* 1984; 1:45-9.
27. Chopra A, Kalkanis A, Judson MA. Biomarkers in sarcoidosis. *Expert Rev Clin Immunol* 2016; 12:1191-208.
28. Grutters JC, Fellrath JM, Mulder L, et al. Serum soluble interleukin-2 receptor measurement in patients with sarcoidosis: a clinical evaluation. *Chest* 2003; 124:186-95.
29. Nakatsu M, Hatabu H, Morikawa K, et al. Large coalescent parenchymal nodules in pulmonary sarcoidosis: "sarcoid galaxy" sign. *AJR Am J Roentgenol* 2002; 178:1389-1393.
30. Keijsers RG, Veltkamp M, Grutters JC. Chest Imaging. *Clin Chest Med* 2015; 36:603-19.
31. Greco FG, Spagnolo P, Muri M, et al. The value of chest radiograph and computed tomography in pulmonary sarcoidosis. *Sarcoidosis Vasc Diffuse Lung Dis* 2014; 31:108-16.
32. Akira M, Kozuka T, Inoue Y, et al. Long-term follow-up CT scan evaluation in patients with pulmonary sarcoidosis. *Chest* 2005; 127:185-91.
33. Keir G, Wells AU. Assessing pulmonary disease and response to therapy: which test? *Semin Respir Crit Care Med* 2010; 31:409-18.
34. Zappala CJ, Desai SR, Copley SJ, et al. Optimal scoring of serial change on chest radiography in sarcoidosis. Sarcoidosis Vasc Diffuse Lung Dis. 2011; 28:130-8.
35. Baughman RP, Drent M, Culver DA, et al. Endpoints for clinical trials of sarcoidosis. Sarcoidosis Vasc Diffuse Lung Dis. 2012; 29:90-8.
36. Judson MA. Corticosteroids in Sarcoidosis. *Rheum Dis Clin North Am* 2016; 42:119-35, ix.

For peer review only

Table 1. Organ involvement in sarcoidosis patients

Organ	No. (%)
Isolated pulmonary involvement	378 (59.4)
Pulmonary sarcoidosis with extrapulmonary involvement	258 (40.6)
Extrathoracic lymph node	147 (23.1)
Skin	86 (13.5)
Eyes	8 (1.3)
Bone/joints	8 (1.3)
Liver	4 (0.6)
Nose	4 (0.6)
Parotid/salivary	3 (0.5)
Spleen	2 (0.3)
Renal	2 (0.3)
Cardiac	1 (0.2)
Muscles	1 (0.2)

Total numbers add up to greater than the total number of patients because 8 patients had more than one organ involvement.

For peer review only

Table 2. Baseline and clinical characteristics of subjects with pulmonary sarcoidosis, with and without extrapulmonary involvement

Parameter	Isolated pulmonary sarcoidosis	Pulmonary sarcoidosis with extrapulmonary involvement	p-value
Subjects, No. (%)	378 (59.4)	258 (40.6)	—
Sex, M/F, No.	260/118	174/84	0.721
Age, y	48.8±10.7	48.2±11.5	0.613
BMI, kg/m^2	23.9±3.3	23.4±3.3	0.033
Smoking history, No. (%)	59 (15.6)	29 (11.2)	0.114
Occupational exposure, No. (%)	33 (8.7)	18 (7.0)	0.424
Symptoms, No. (%)	277 (73.3)	181 (70.2)	0.467
Cough, No. (%)	231 (83.4)	142 (78.5)	0.127
Chest congestion, No. (%)	80 (28.9)	50 (27.6)	0.548
Dyspnea, No. (%)	50 (18.1)	36 (19.9)	0.793
Chest pain, No. (%)	39 (14.1)	19 (10.5)	0.204
Fatigue, No. (%)	23 (8.3%)	30 (16.6%)	0.013
Fever, No. (%)	18 (6.5)	10 (5.5)	0.593
Scadding stage			
Stage 1, No. (%)	128（33.9）	67（25.9）	0.034
Stage 2, No. (%)	232（61.4）	180（69.8）	0.0296
Stage 3, No. (%)	18（4.7）	11（4.3）	0.7674

Data are presented as mean±SD or %. BMI: body mass index.

For peer review only

Table 3. Serological indicators of subjects with pulmonary sarcoidosis, with and without extrapulmonary involvement

Parameter	Isolated pulmonary sarcoidosis	Pulmonary sarcoidosis with extrapulmonary involvement	p-value
CRP, mg/L	4.0±4.2	3.8±3.0	0.547
TB-AB, No. %	22 (5.8)	20 (7.8)	0.335
SIL-2R, U/ml	1151±842	1163±798	0.32
UC, mmol/24h	6.09±2.94	6.08±2.65	0.117
SC, mmol/L	2.39±0.13	2.42±0.15	0.022
SACE, IU/L	69.7±38.7	79.0±46.9	0.016
ALP, IU/L	86.4±33.4	84.7±33.4	0.259
ALB, g/L	40.16±4.02	40.81±3.86	0.8
GLB, g/L	27.96±5.34	30.14±5.67	0.013
A/G	1.47±0.28	1.42±0.27	0.064
UA,umol/L	309.4±85.4	308.6±99.3	0.728
Seroglobulin β	11.23±1.71	11.30±1.43	0.278
Seroglobulin γ	17.72±3.93	18.40±4.13	0.084
WBC,10^9/L	6.2±2.1	5.7±2.0	0.377
ESR,mm/H	15.8±10.7	15.8±9.3	0.288

Data are presented as mean±SD or %. CRP: C-reactive protein; TB-AB: tuberculosis antibody; SIL-2R: soluble interleukin 2 receptor; UC: urinary calcium; SC: serum calcium; SACE: serum angiotensin converting enzyme; ALP: alkaline phosphatase; ALB: albumin; GLB: globulin; A/G: albumin/globulin ratio; UA: blood uric acid; WBC: white blood count; ESR: erythrocyte sedimentation rate.

For peer review only

Table 4. Pulmonary function parameters of subjects with pulmonary sarcoidosis, with and without extrapulmonary involvement

Parameter	Isolated pulmonary sarcoidosis	Pulmonary sarcoidosis with extrapulmonary involvement	p-value
VC, L	3.04±0.75	3.09±0.82	0.327
FVC, L	3.03±0.75	3.07±0.81	0.309
FVC % pred	97.5 ± 15.6	96.8 ± 17.2	0.305
FEV1, L	3.03±0.75	3.07±0.81	0.21
FEV1 % pred	88.61 ± 15.08	88.89 ± 16.30	0.452
FEV1/FVC	78.62±7.74	78.94±8.52	0.072
RV, L	1.84±0.43	1.83±0.55	0.317
TLC, L	4.86±0.93	4.89±1.03	0.972
RV/TLC	38.51 ± 7.66	37.73 ± 9.16	0.137
DLCO, ml/min/mmHg	21.1 ± 4.8	20.6 ± 5.2	0.487
Airflow limitation, No. (%)	160 (42.3)	109 (42.2)	0.984
Volume restriction, No. (%)	27 (7.1)	12 (4.7)	0.198

Data are presented as mean±SD or %. VC: vital capacity; FVC: forced vital capacity; FEV1: forced expiratory volume in 1 s; RV: residual volume; TLC: total lung capacity; DLCO: diffusion capacity for carbon monoxide of lung.

For peer review only

Table 5. Factors associated with deteriorating HRCT findings in all subjects according to the logistic regression analysis

Parameter	OR (95% CI)	p-value
Extrapulmonary involvement	0.516（0.232-1.147）	0.104
Corticosteroid therapy	0.941（0.42-2.11）	0.884
FVC% pre	0.998（0.978-1.108）	0.843
FEV1/FVC ratio	0.998（0.954-1.043）	0.928

FVC: forced vital capacity; FEV1: forced expiratory volume in 1 s.

Legend of figures

Figure 1. HRCT findings in sarcoidosis patients. (a) Shows pulmonary micronodules at the time of initial hospitalization; (b) with pulmonary involvement during follow-up. (c) Shows enlarged mediastinal lymph node at the time of initial hospitalization; (d) The mediastinal lymph node became larger during follow-up.

Figure 2. (a) Pulmonary sarcoidosis with or without extrapulmonary involvement; (b) with or without corticosteroid therapy; (c) forced vital capacity (FVC) % predicted; (d) forced expiratory volume in 1 s (FEV1) / FVC ratio of patients who had deteriorating chest radiograph findings and patients who did not have deteriorating chest radiograph findings.

For peer review only

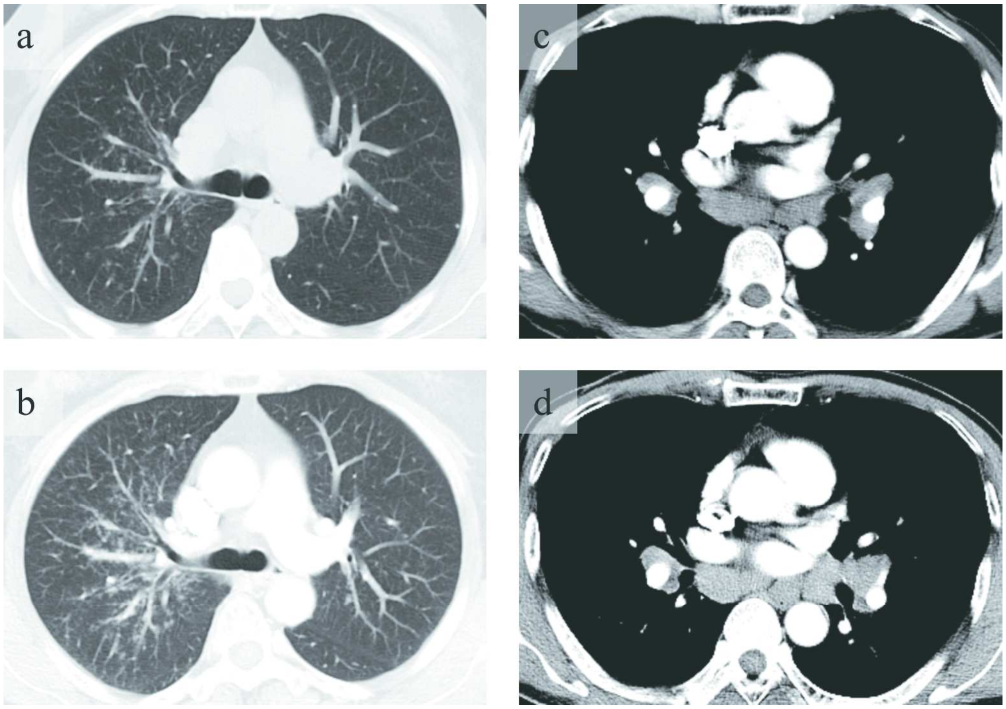

Figure 1. HRCT findings in sarcoidosis patients. (a) Shows pulmonary micronodules at the time of initial hospitalization; (b) with pulmonary involvement during follow-up. (c) Shows enlarged mediastinal lymph node at the time of initial hospitalization; (d) The mediastinal lymph node became larger during follow-up.

268x188mm (300 x 300 DPI)

For peer review only - http://bmjopen.bmj.com/site/about/guidelines.xhtml

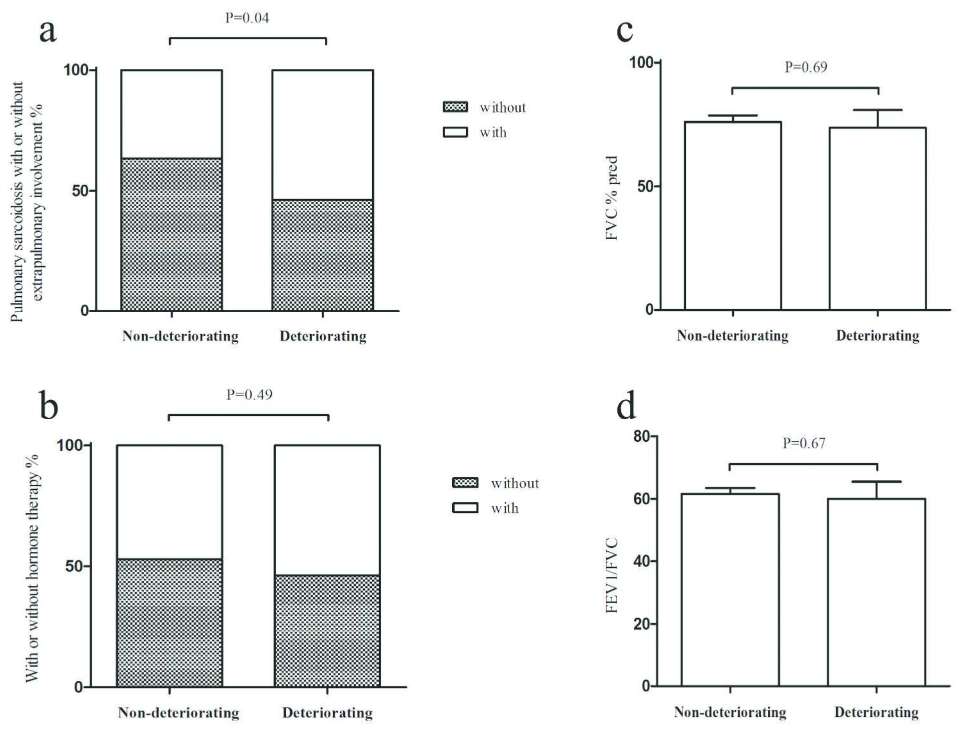

Figure 2. (a) Pulmonary sarcoidosis with or without extrapulmonary involvement; (b) with or without corticosteroid therapy; (c) forced vital capacity (FVC) % predicted; (d) forced expiratory volume in 1 s (FEV1) / FVC ratio of patients who had deteriorating chest radiograph findings and patients who did not have deteriorating chest radiograph findings.

256x190mm (300 x 300 DPI)

For peer review only

STROBE Statement—Checklist of items that should be included in reports of ***cross-sectional studies***

	Item No	Recommendation
**Title and abstract**	1	(*a*) Indicate the study's design with a commonly used term in the title or the abstract P1
		(*b*) Provide in the abstract an informative and balanced summary of what was done and what was found P2
**Introduction**		
Background/rationale	2	Explain the scientific background and rationale for the investigation being reported P4
Objectives	3	State specific objectives, including any prespecified hypotheses P4
**Methods**		
Study design	4	Present key elements of study design early in the paper P4
Setting	5	Describe the setting, locations, and relevant dates, including periods of recruitment, exposure, follow-up, and data collection P4
Participants	6	(*a*) Give the eligibility criteria, and the sources and methods of selection of participants P4
Variables	7	Clearly define all outcomes, exposures, predictors, potential confounders, and effect modifiers. Give diagnostic criteria, if applicable P4
Data sources/ measurement	8*	For each variable of interest, give sources of data and details of methods of assessment (measurement). Describe comparability of assessment methods if there is more than one group P4 P5
Bias	9	Describe any efforts to address potential sources of bias P7
Study size	10	Explain how the study size was arrived at P4
Quantitative variables	11	Explain how quantitative variables were handled in the analyses. If applicable, describe which groupings were chosen and why P4 P5
Statistical methods	12	(*a*) Describe all statistical methods, including those used to control for confounding P5
		(*b*) Describe any methods used to examine subgroups and interactions P5
		(*c*) Explain how missing data were addressed NONE
		(*d*) If applicable, describe analytical methods taking account of sampling strategy P5
		(*e*) Describe any sensitivity analyses P5
**Results**		
Participants	13*	(a) Report numbers of individuals at each stage of study—eg numbers potentially eligible, examined for eligibility, confirmed eligible, included in the study, completing follow-up, and analysed P5 P6
		(b) Give reasons for non-participation at each stage NONE
		(c) Consider use of a flow diagram NONE
Descriptive data	14*	(a) Give characteristics of study participants (eg demographic, clinical, social) and information on exposures and potential confounders P5
		(b) Indicate number of participants with missing data for each variable of interest P5 P6
Outcome data	15*	Report numbers of outcome events or summary measures P5 P6
Main results	16	(*a*) Give unadjusted estimates and, if applicable, confounder-adjusted estimates and their precision (eg, 95% confidence interval). Make clear which confounders were adjusted for and why they were included P6
		(*b*) Report category boundaries when continuous variables were categorized P5 P6

For peer review only - http://bmjopen.bmj.com/site/about/guidelines.xhtml

		(*c*) If relevant, consider translating estimates of relative risk into absolute risk for a meaningful time period P6
Other analyses	17	Report other analyses done—eg analyses of subgroups and interactions, and sensitivity analyses P5 P6
**Discussion**		
Key results	18	Summarise key results with reference to study objectives P6 P7
Limitations	19	Discuss limitations of the study, taking into account sources of potential bias or imprecision. Discuss both direction and magnitude of any potential bias P7
Interpretation	20	Give a cautious overall interpretation of results considering objectives, limitations, multiplicity of analyses, results from similar studies, and other relevant evidence P6 P7
Generalisability	21	Discuss the generalisability (external validity) of the study results P6 P7
**Other information**		
Funding	22	Give the source of funding and the role of the funders for the present study and, if applicable, for the original study on which the present article is based P8

*Give information separately for exposed and unexposed groups.

For peer review only

BMJ Open

Pulmonary sarcoidosis with and without extrapulmonary involvement : a cross-sectional and observational study in China

Journal:	*BMJ Open*
Manuscript ID	bmjopen-2017-018865.R2
Article Type:	Research
Date Submitted by the Author:	18-Jan-2018
Complete List of Authors:	li, chengwei; Shanghai Pulmonary Hospital, Tongji University School of Medicine, Department of Respiratory and Critical Care Medicine<br>tao, rujia; Shanghai Pulmonary Hospital, Tongji University School of Medicine, Department of Respiratory and Critical Care Medicine<br>zou, danfeng; Shanghai Pulmonary Hospital, Tongji University School of Medicine, Clinic and Research Center of Tuberculosis, Shanghai Key Lab of Tuberculosis<br>li, manhui; Shanghai Pulmonary Hospital, Tongji University School of Medicine, Department of Respiratory and Critical Care Medicine<br>xu, xin; Shanghai Pulmonary Hospital, Tongji University School of Medicine, Department of Respiratory and Critical Care Medicine<br>cao, weijun; Tongji University Affiliated Shanghai Pulmonary Hospital, Department of Respiratory Medicine
**Primary Subject Heading**:	Respiratory medicine
Secondary Subject Heading:	Respiratory medicine, Radiology and imaging
Keywords:	sarcoidosis, extrapulmonary involvement, fatigue, prognosis

SCHOLARONE™
Manuscripts

Pulmonary sarcoidosis with and without extrapulmonary involvement：a cross-sectional and observational study in China

Cheng-Wei Li[a#], Ru-Jia Tao[b#], Dan-Feng Zou[c#], Man-Hui Li[b], Xin Xu[b], Wei-Jun Cao[b]

Institution:
a: Department of Respiratory and Critical Care Medicine, Shanghai Pulmonary Hospital, Soochow University, Suzhou 215006, China
b: Department of Respiratory and Critical Care Medicine, Shanghai Pulmonary Hospital, Tongji University School of Medicine, Shanghai 200433, China
c: Clinic and Research Center of Tuberculosis, Shanghai Key Lab of Tuberculosis, Shanghai Pulmonary Hospital, Tongji University School of Medicine, Shanghai 200433, China
#: These authors contributed equally.

Corresponding author: Wei-Jun Cao, Department of Respiratory and Critical Care Medicine, Shanghai Pulmonary Hospital, Tongji University School of Medicine, No. 507 Zhengmin Road, Shanghai, 200433, China. E-mail: weijuncao@126.com

Word count: 2,091 words.

For peer review only

ABSTRACT

Objectives: Sarcoidosis is a multisystem disease characterized by the formation of granulomas within various organs, mainly the lungs. Several studies from different countries have been undertaken to investigate sarcoidosis with extrapulmonary involvement but from China. The objective of this study is to investigate a comparative clinical analysis in pulmonary sarcoidosis patients with and without extrapulmonary involvement from China.

Methods: Data from inpatients diagnosed with sarcoidosis at Shanghai Pulmonary Hospital (Shanghai, China) between January 2009 and December 2014 were retrospectively collected and analyzed. Six hundred and thirty-six biopsy proven sarcoidosis patients were included in the study, including 378 isolated pulmonary sarcoidosis and 258 pulmonary sarcoidosis plus extrapulmonary involvement.

Results: Two hundred and fifty eight (40.6%) pulmonary sarcoidosis patients had extrapulmonary involvement. Extrapulmonary localizations were detected mostly in extrathoracic lymph nodes (n=147) and skin (n=86). Statistically significant differences were demonstrated between patients with pulmonary sarcoidosis plus extrapulmonary involvement and patients with isolated pulmonary sarcoidosis for fatigue (16.6% vs. 8.3%, $p<0.05$), serum angiotensin-converting enzyme (SACE) levels (79.0±46.9 IU/L vs. 69.7±38.7 IU/L, $p<0.05$), and high-resolution computed tomography (HRCT) findings (53.8% vs. 46.2%, $p<0.05$).

Conclusions:Extrapulmonary involvement is common in pulmonary sarcoidosis patients, with the most common sites being extrathoracic lymph nodes and skin. Sarcoidosis patients with extrapulmonary involvement are more symptomatic (fatigue), have higher SACE levels and more deteriorating HRCT findings, to which clinicians should pay attention.

Keywords: sarcoidosis; extrapulmonary involvement; fatigue; prognosis

For peer review only

Article summary

Strengths and limitations of this study

1. The present study provides a large number of pulmonary sarcoidosis patients with and without extrapulmonary involvement from China.
2. The sarcoidosis patients had a long term follow-up (24-42 months) on chest HRCT.
3. There is a risk of selection bias due to the retrospective design.

For peer review only

INTRODUCTION

Sarcoidosis is a granulomatous disease of unknown etiology. It is characterized by the formation of immune granulomas in many involved organs.[1,2] Sarcoidosis is a worldwide disease, with its prevalence ranging from 4.7 to 64 in 100 000, and its incidence ranging from 1.0 to 35.5 in 100 000 per year.[3] However, limited epidemiological data in China is available.

Sarcoidosis is a multi-system disease. Although the lungs are most commonly involved, pulmonary sarcoidosis is often accompanied by the involvement of extrapulmonary organs,[1,4] such as the skin, eyes, and heart that are significantly affected.[5] A Case Control Etiologic Study of Sarcoidosis (ACCESS) showed that among 736 patients with sarcoidosis, 95% of the patients had pulmonary involvement, and 368 out of these 736 patients (50%) had concomitant extrapulmonary involvement.[6] Extrapulmonary involvement can be the major presentation of the disease and sometimes may be life-threatening.[5,7] Moreover, the assessment of pulmonary and extrapulmonary organ involvement is important for clinical treatment decisions.[8]

It is well known that the features of sarcoidosis have varied widely among various populations around the world. To date, several studies from different countries have been undertaken to investigate sarcoidosis with extrapulmonary involvement. However, there is limited information in Chinese sarcoidosis patients. Therefore, the purpose of this study is to compare the clinical characteristics and prognosis of pulmonary sarcoidosis patients with and without extrapulmonary involvement from China.

METHODS

Data were retrospectively collected from inpatients diagnosed with sarcoidosis at the Shanghai Pulmonary Hospital (Shanghai, China) between January 2009 and December 2014. Patients who had not received a chest radiograph or chest high resolution computed tomography (HRCT) scan examination or who had not received tissue biopsy were excluded. All aspects of the study were performed in accordance with relevant guidelines and regulations.

Diagnosis of sarcoidosis is based on standardized criteria,[1] which detailed the features of sarcoidosis, including clinical and radiological presentation, and pathological findings (noncaseating epithelioid granulomas), as well as evidence of no alternative diseases (infections, particularly tuberculosis; occupationally induced, environmentally induced, and drug-induced granulomatosis; common variable immune deficiency; Blau's syndrome; sarcoid-like reactions in cancers and lymphomas, and other idiopathic granulomatosis).[3]

Organ involvement was determined and classified in each patient in accordance with criteria proposed in the ACCESS formulation,[9] based upon clinical assessment and widely available tests, including the evolution of electrocardiography, positron emission tomography (PET) scans as well as magnetic resonance imaging (MRI) that can improve the detection of the disease. According to the organs affected by sarcoidosis, patients were divided into two groups: isolated pulmonary sarcoidosis and pulmonary sarcoidosis with extrapulmonary involvement.

The following variables were obtained from the medical records: general and anthropometric information, symptoms, chest radiographs or CT films, serological indicators, and pulmonary function parameters. The chest radiographs or CT films were read at each site by two investigators; the findings were evaluated and staged according to the modified Scadding criteria.[10] Pulmonary function studies were performed and a percentage of the subject's predicted value was calculated,[11] airflow limitation was defined as an forced expiratory volume in one second/forced vital

For peer review only

capacity ratio (FEV1/FVC ratio) below the 5th percentile of the predicted value; and the restrictive pattern was defined as forced vital capacity (FVC) below the predicted lower limit, with a normal FEV1/FVC ratio. The patients were followed up on chest HRCT at the outpatient clinic of Shanghai Pulmonary Hospital. The HRCT results were assessed by two independent radiologists. The deteriorating chest HRCT findings (an increase in extent compared with the previous HRCT results) were recorded in patients after their discharge from the hospital (figure 1). Written informed consent was obtained from all of the patients. This study was approved by the Ethics Committee of Shanghai Pulmonary Hospital. All aspects of the study were performed in accordance with relevant guidelines and regulations.

Statistical Analysis

Quantitative variables were expressed as the mean± standard deviation (SD) and qualitative variables were expressed as absolute numbers and percentages. The Kolmogorov–Smirnov test was used to analyze the distribution of variables. In the bivariate analysis, the t-test for independent variables was used to analyze normally distributed variables and the Mann-Whitney U-test was used to analyze non-normally distributed variables. Qualitative variables were compared using the Chi-squared test. A logistic regression model was used to determine the factors that were independently associated with deteriorating chest HRCT findings. The variables that presented statistically significant differences ($p<0.05$) in the bivariate analysis and were of clinical interest were included as the independent variables in the model. The odds ratio (OR) and 95% confidence interval were calculated for the independent variables. Statistical analysis was performed using SPSS 22.0 package software with a value of $p <0.05$ considered statistically significant, and graphs were drawn using GraphPad Prism 5 Software.

Results

A total of 636 biopsy proven sarcoidosis patients (378 isolated pulmonary sarcoidosis and 258 pulmonary sarcoidosis with extrapulmonary involvement) were included in the study. Among these 636 patients, 298 responded fully to the follow-up study on HRCT (182 isolated pulmonary sarcoidosis and 116 pulmonary sarcoidosis with extrapulmonary involvement), the median follow-up time after discharge was 30 months (ranging from 24 to 42 months). Thirty-nine patients had deteriorating chest HRCT findings after their discharge from the hospital.

The organ involvement and baseline characteristics of the subjects were shown in tables 1 and 2; the serological and pulmonary function parameters were shown in tables 3 and 4, respectively. Extrapulmonary localizations were detected mostly in extrathoracic lymph nodes (n=147) and skin (n=86). There were statistical differences in body mass index (BMI), fatigue, Scadding stage distribution, serum calcium (SC), SACE and globulin (GLB) levels. The airflow limitation was observed in 42.3% of patients with isolated pulmonary sarcoidosis patients and 42.2% of patients with pulmonary sarcoidosis and extrapulmonary involvement with no statistical significance between these two groups (p=0.984). Similar result was seen in volume restriction between these two groups (7.1% vs. 4.7%, p=0.198).

The differential characteristics of patients with or without deteriorating chest HRCT findings were shown in figure 2. Pulmonary sarcoidosis patients with extrapulmonary involvement had more deteriorating HRCT findings than patients with isolated pulmonary sarcoidosis (21 vs. 18, p=0.04). Even though corticosteroid therapy, FVC% predicted and FEV1/FVC ratio were not statistically significant, we set these parameters as independent variables in the logistic regression model to investigate whether these factors had influence on prognosis of HRCT.

The OR value and 95% confidence intervals of variables related to deteriorating HRCT findings in all patients were illustrated in Table 5. However, the extrapulmonary involvement, corticosteroid therapy, FVC% predicted, and FEV1/FVC ratio were irrelevant to the risk of deteriorating HRCT findings in these patients.

Discussion

The current study aimed to retrospectively compare the clinical characteristics of pulmonary sarcoidosis patients with and without extrapulmonary involvement as well as their prognosis on HRCT. So far, several studies conducted by different countries have been undertaken to investigate extrapulmonary sarcoidosis except for China. Since clinical features of sarcoidosis have varied among various populations around the world, our study would provide valuable information and therefore, may help facilitate the prognosis and management for Chinese sarcoidosis patients.

The prevalence of extrapulmonary sarcoidosis involvement varies among populations. Based on our study, we found that 40.6% pulmonary sarcoidosis patients had extrapulmonary involvement, which was consistent with previous study in which 30–50% of patients showed extrapulmonary disease localisations.[7] The most common sites found in our study were extrathoracic lymph nodes and skin, which is similar to studies from Turkey,[12,13] but differs from other researches. Skin lesions are divided into two categories: specific and nonspecific. In this study most of skin lesions are specific lesions that demonstrate granulomatous inflammation on biopsy. The rest are nonspecific lesions that show no granulomatous inflammation, such as erythema nodosum. ACCESS demonstrated that Caucasians more frequently had a sarcoidosis-related calcium metabolism disorder;[14] Finnish and Japanese patients were found to have higher rates of cardiac and eye involvement;[15] Puerto Ricans were common with Lupus pernio skin lesions; and Europeans had more erythema nodosum lesions.[1] Extrapulmonary sarcoidosis may be more frequent in females, illustrated by a health maintenance organization database,[16] as well as a study from Beijing Tongren Hospital (Beijing, China).[17] However, we did not find the difference between gender in this study.

The most common symptom of mediastinal–pulmonary manifestations of sarcoidosis is persistent cough. Dyspnea is rare in the early stages, but more frequent later on.[3] Another symptom is fatigue that occurs in up to 70% of patients, which can be measured and monitored with a validated fatigue assessment scale,[18] which could predict quality of life.[19] Previous research showed that patients with pulmonary and extrapulmonary sarcoidosis were more fatigued and more dyspneic than those with pulmonary involvement alone, which demonstrated differences in the severity of symptoms.[20] In our study, a portion of patients had no symptoms, so we analyzed the proportion of various complaint, and found that extrapulmonary involvement patients had higher proportion of fatigue than isolated pulmonary sarcoidosis patients (16.6% vs. 8.3%, p=0.013). Neither cough nor dyspnea was found to be different between the two groups in our study.

Of note, in our study we found pulmonary sarcoidosis patients with extrapulmonary involvement had higher level of serum ACE (SACE) than patients with isolated pulmonary sarcoidosis (79.0±46.9 vs. 69.7±38.7 IU/L, p=0.016). Angiotensin convertase enzyme (ACE) is a caboxypeptidase enzyme that converts angiotensin I into angiotensin II,[21] which is produced in the epitheliod cell of the sarcoid granuloma.[22] SACE level, especially the amount of ACE activity, has been proposed as a biomarker that reflects the burden of granulomas.[23,24] Previous research also revealed that SACE level had correlated with extrapulmonary organs involvement and

For peer review only

overall disease activity.[25,26] SACE is related to the various polymorphisms of the ACE gene, so different races may have significant differences in their SACE levels.[27] Another significant biomarker in sarcoidosis is soluble interleukin 2 receptor (sIL-2R). A previous study showed that sIL-2R was higher in patients with extrapulmonary sarcoidosis than those with isolated pulmonary sarcoidosis.[28] However, in our study, there was no difference in sIL-2R concentration (1151±842 vs. 1163±798 U/ml in patients with and without extrapulmonary involvement, respectively, $p=0.32$). Further studies may be necessary to demonstrate the connection between serum biomarkers and differences in races and phenotypes.

Using chest radiograph or CT to view the internal structure is the most common imaging biomarker for evaluating sarcoidosis. The sarcoidosis granulomas can be detected with plain radiographic or computed tomography radiographic techniques.[29] In our study, isolated pulmonary sarcoidosis patients had higher proportion in stage I (n=128, $p<0.05$), while sarcoidosis patients with extrapulmonary involvement had higher proportion in stage II (n=180, $p<0.05$). Even though Scadding criteria provided useful prognostic information, these stages do not necessarily progress or regress from one to another.[30] CT is useful in assessing disease extent;[31] HRCT may also provide prognostic information.[32,33] However, CJ. Zappala *et al* found that change in radiographic extent was more applicable to routine monitoring in sarcoidosis than change in radiographic stage.[34] So we used deteriorating chest HRCT findings (an increase in extent compared with the previous HRCT results) as prognosis of sarcoidosis. FVC is the most common end point in pulmonary sarcoidosis trials;[35] significant airway obstruction is also associated with sarcoidosis via different mechanisms.[27] Corticosteroids are considered the drug for the treatment of almost all forms of sarcoidosis.[36] Even though these three factors were not statistically significant between patients with and without deteriorating HRCT findings, we still considered and included them as independent variables in the logistic regression model. However, we did not find relevant risk factors of deteriorating HRCT findings in these patients.

There are several limitations in this study. Firstly, there is a risk of selection bias due to the retrospective design. Therefore, we conducted a cross-sectional and observational study which might reduce the risk of selection bias. Secondly, we conducted this study more from a pulmonary perspective. Since sarcoidosis is a multi-system disease, a more comprehensive study could be performed to investigate other parameters related to sarcoidosis. Thirdly, to date, no reliable prognostic biomarkers have been identified. Based on the previous research,[34] we used deteriorating chest HRCT findings as a prognostic factor of sarcoidosis. Further studies are needed to demonstrate potential biomarkers that would predict the prognosis. Finally, the generalisability of our findings may be limited due to the number of single-center that participated in our study, hence, our findings require additional validation, including data from other languages and other institutions.

In summary, our results suggest that extrapulmonary involvement is common in pulmonary sarcoidosis patients, with the most common sites being extrathoracic lymph nodes and skin. Sarcoidosis patients with extrapulmonary involvement have more complaint of fatigue, higher SACE levels and more deteriorating HRCT findings than those with isolated pulmonary sarcoidosis, to which clinicians should pay attention.

Data Sharing Statement: No additional data available.

Contributorship Statement: Wei-Jun Cao has full access to all of the data in the

For peer review only

study and takes responsibility for the integrity of the data and the accuracy of the data analysis. Cheng-Wei Li, Ru-Jia Tao and Dan-Feng Zou are joint first authors. Wei-Jun Cao and Xin Xu contributed to the study concept and design; Ru-Jia Tao and Man-Hui Li contributed to data collection and interpretation; Cheng-Wei Li and Dan-Feng Zou performed the statistical analyses; finally all authors discussed data results and approved this submission.

Financial support: This research was funded by the National Key Research and Development Plan of Precision Medicine Project (Grant No. 2016YFC0905700).

Role of the Sponsors: The funding source had no role in the design and conduct of the study; collection, management, analysis, and interpretation of the data; preparation, review, or approval of the manuscript; and decision to submit the manuscript for publication.

Conflict of Interest: The authors have declared that no conflict of interest exists.

Reference

1. Hunninghake GW, Costabel U, Ando M, et al. ATS/ERS/WASOG statement on sarcoidosis. American Thoracic Society/European Respiratory Society/World Association of Sarcoidosis and other Granulomatous Disorders. *Sarcoidosis Vasc Diffuse Lung Dis* 1999; 16:149-73.
2. Baughman RP, Lower EE, du Bois RM. Sarcoidosis. *Lancet* 2003; 361:1111-8.
3. Valeyre D, Prasse A, Nunes H, et al. Sarcoidosis. *Lancet* 2014; 383:1155-67.
4. Bauer MP, Brouwer PA, Smit VT, et al. The challenges of extrapulmonary presentations of sarcoidosis: A case report and review of diagnostic strategies. *Eur J Intern Med* 2007; 18:152-4.
5. Rao DA, Dellaripa PF. Extrapulmonary manifestations of sarcoidosis. *Rheum Dis Clin North Am* 2013; 39:277-97.
6. Baughman R, Teirstein A, Ma, Rossman M, et al. Clinical characteristics of patients in a case control study of sarcoidosis. *American Journal of Respiratory & Critical Care Medicine* 2001; 164:1885-9.
7. Judson MA. Extrapulmonary sarcoidosis. *Seminars in Respiratory Infections* 2007; 28:83-101.
8 .Judson MA, Baughman RP, Teirstein AS, et al. Defining organ involvement in sarcoidosis: the ACCESS proposed instrument. ACCESS Research Group. A Case Control Etiologic Study of Sarcoidosis. *Sarcoidosis Vasc Diffuse Lung Dis* 1999; 16:75-86.
9. Judson MA, Costabel U, Drent M, et al. The WASOG Sarcoidosis Organ Assessment Instrument: An update of a previous clinical tool. *Sarcoidosis Vasc Diffuse Lung Dis* 2014; 31:19-27.
10. Scadding JG. Prognosis of intrathoracic sarcoidosis in England. A review of 136 cases after five years' observation. *Br Med J* 1961; 2:1165-72.
11. Hankinson JL, Odencrantz JR, Fedan KB. Spirometric reference values from a sample of the general U.S. population. *Am J Respir Crit Care Med* 1999; 159:179-87.
12. Okumus G, Musellim B, Cetinkaya E, et al. Extrapulmonary involvement in patients with sarcoidosis in Turkey. *Respirology* 2011; 16:446-50.
13. Zurkova M, Kolek V, Tomankova T, et al. Extrapulmonary involvement in patients with sarcoidosis and comparison of routine laboratory and clinical data to pulmonary involvement. *Biomed Pap Med Fac Univ Palacky Olomouc Czech Repub* 2014; 158:613-20.
14. Baughman RP, Teirstein AS, Judson MA, et al. Clinical characteristics of patients in a case control study of sarcoidosis. *Am J Respir Crit Care Med* 2001; 164:1885-9.
15. Pietinalho A, Ohmichi M, Hirasawa M, et al. Familial sarcoidosis in Finland and Hokkaido, Japan--a comparative study. *Respir Med* 1999; 93:408-12.
16. Rybicki BA, Major M, Popovich J, Jr., et al. Racial differences in sarcoidosis incidence: a 5-year study in a health maintenance organization. *Am J Epidemiol* 1997; 145:234-41.
17. Jin JM, Sun YC, Zhuo J, et al. A comparative clinical analysis of thoracic sarcoidosis and multi-organ sarcoidosis. *Zhonghua Jie He He Hu Xi Za Zhi* 2011; 34:914-8.
18. De Vries J, Michielsen H, Van Heck GL, et al. Measuring fatigue in sarcoidosis: the Fatigue Assessment Scale (FAS). *Br J Health Psychol* 2004; 9:279-91.
19. Michielsen HJ, Peros-Golubicic T, Drent M, et al. Relationship between symptoms and quality of life in a sarcoidosis population. *Respiration* 2007; 74:401-5.
20. Gvozdenovic BS, Mihailovic-Vucinic V, Ilic-Dudvarski A, et al. Differences in symptom severity and health status impairment between patients with pulmonary and pulmonary plus extrapulmonary

For peer review only

sarcoidosis. *Respir Med* 2008; 102:1636-42.
21. Ryan JW, Smith U, Niemeyer RS. Angiotensin I: metabolism by plasma membrane of lung. *Science* 1972; 176:64-6.
22. Sheffield EA. Pathology of sarcoidosis. *Clin Chest Med* 1997; 18:741-54.
23. Lynch JP, Kazerooni EA, Gay SE. Pulmonary sarcoidosis. *Clin Chest Med* 1997; 18:755-85.
24. Gilbert S, Steinbrech DS, Landas SK, et al. Amounts of angiotensin-converting enzyme mRNA reflect the burden of granulomas in granulomatous lung disease. *Am Rev Respir Dis* 1993; 148:483-6.
25. Klech H, Kohn H, Kummer F, et al. Assessment of activity in Sarcoidosis. Sensitivity and specificity of 67Gallium scintigraphy, serum ACE levels, chest roentgenography, and blood lymphocyte subpopulations. *Chest* 1982; 82:732-8.
26. Selroos OB. Value of biochemical markers in serum for determination of disease activity in sarcoidosis. *Sarcoidosis* 1984; 1:45-9.
27. Chopra A, Kalkanis A, Judson MA. Biomarkers in sarcoidosis. *Expert Rev Clin Immunol* 2016; 12:1191-208.
28. Grutters JC, Fellrath JM, Mulder L, et al. Serum soluble interleukin-2 receptor measurement in patients with sarcoidosis: a clinical evaluation. *Chest* 2003; 124:186-95.
29. Nakatsu M, Hatabu H, Morikawa K, et al. Large coalescent parenchymal nodules in pulmonary sarcoidosis: "sarcoid galaxy" sign. *AJR Am J Roentgenol* 2002; 178:1389-1393.
30. Keijsers RG, Veltkamp M, Grutters JC. Chest Imaging. *Clin Chest Med* 2015; 36:603-19.
31. Greco FG, Spagnolo P, Muri M, et al. The value of chest radiograph and computed tomography in pulmonary sarcoidosis. *Sarcoidosis Vasc Diffuse Lung Dis* 2014; 31:108-16.
32. Akira M, Kozuka T, Inoue Y, et al. Long-term follow-up CT scan evaluation in patients with pulmonary sarcoidosis. *Chest* 2005; 127:185-91.
33. Keir G, Wells AU. Assessing pulmonary disease and response to therapy: which test? *Semin Respir Crit Care Med* 2010; 31:409-18.
34. Zappala CJ, Desai SR, Copley SJ, et al. Optimal scoring of serial change on chest radiography in sarcoidosis. Sarcoidosis Vasc Diffuse Lung Dis. 2011; 28:130-8.
35. Baughman RP, Drent M, Culver DA, et al. Endpoints for clinical trials of sarcoidosis. Sarcoidosis Vasc Diffuse Lung Dis. 2012; 29:90-8.
36. Judson MA. Corticosteroids in Sarcoidosis. *Rheum Dis Clin North Am* 2016; 42:119-35, ix.

For peer review only

Table 1. Organ involvement in sarcoidosis patients

Organ	No. (%)
Isolated pulmonary involvement	378 (59.4)
Pulmonary sarcoidosis with extrapulmonary involvement	258 (40.6)
Extrathoracic lymph node	147 (23.1)
Skin	86 (13.5)
Eyes	8 (1.3)
Bone/joints	8 (1.3)
Liver	4 (0.6)
Nose	4 (0.6)
Parotid/salivary	3 (0.5)
Spleen	2 (0.3)
Renal	2 (0.3)
Cardiac	1 (0.2)
Muscles	1 (0.2)

Total numbers add up to greater than the total number of patients because 8 patients had more than one organ involvement.

For peer review only

Table 2. Baseline and clinical characteristics of subjects with pulmonary sarcoidosis, with and without extrapulmonary involvement

Parameter	Isolated pulmonary sarcoidosis	Pulmonary sarcoidosis with extrapulmonary involvement	p-value
Subjects, No. (%)	378 (59.4)	258 (40.6)	—
Sex, M/F, No.	260/118	174/84	0.721
Age, y	48.8±10.7	48.2±11.5	0.613
BMI, kg/m^2	23.9±3.3	23.4±3.3	0.033
Smoking history, No. (%)	59 (15.6)	29 (11.2)	0.114
Occupational exposure, No. (%)	33 (8.7)	18 (7.0)	0.424
Symptoms, No. (%)	277 (73.3)	181 (70.2)	0.467
Cough, No. (%)	231 (83.4)	142 (78.5)	0.127
Chest congestion, No. (%)	80 (28.9)	50 (27.6)	0.548
Dyspnea, No. (%)	50 (18.1)	36 (19.9)	0.793
Chest pain, No. (%)	39 (14.1)	19 (10.5)	0.204
Fatigue, No. (%)	23 (8.3%)	30 (16.6%)	0.013
Fever, No. (%)	18 (6.5)	10 (5.5)	0.593
Scadding stage			
Stage 1, No. (%)	128（33.9）	67（25.9）	0.034
Stage 2, No. (%)	232（61.4）	180（69.8）	0.0296
Stage 3, No. (%)	18（4.7）	11（4.3）	0.7674

Data are presented as mean±SD or %. BMI: body mass index.

For peer review only

Table 3. Serological indicators of subjects with pulmonary sarcoidosis, with and without extrapulmonary involvement

Parameter	Isolated pulmonary sarcoidosis	Pulmonary sarcoidosis with extrapulmonary involvement	p-value
CRP, mg/L	4.0±4.2	3.8±3.0	0.547
TB-AB, No. %	22 (5.8)	20 (7.8)	0.335
SIL-2R, U/ml	1151±842	1163±798	0.32
UC, mmol/24h	6.09±2.94	6.08±2.65	0.117
SC, mmol/L	2.39±0.13	2.42±0.15	0.022
SACE, IU/L	69.7±38.7	79.0±46.9	0.016
ALP, IU/L	86.4±33.4	84.7±33.4	0.259
ALB, g/L	40.16±4.02	40.81±3.86	0.8
GLB, g/L	27.96±5.34	30.14±5.67	0.013
A/G	1.47±0.28	1.42±0.27	0.064
UA,umol/L	309.4±85.4	308.6±99.3	0.728
Seroglobulin β	11.23±1.71	11.30±1.43	0.278
Seroglobulin γ	17.72±3.93	18.40±4.13	0.084
WBC,10^9/L	6.2±2.1	5.7±2.0	0.377
ESR,mm/H	15.8±10.7	15.8±9.3	0.288

Data are presented as mean±SD or %. CRP: C-reactive protein; TB-AB: tuberculosis antibody; SIL-2R: soluble interleukin 2 receptor; UC: urinary calcium; SC: serum calcium; SACE: serum angiotensin converting enzyme; ALP: alkaline phosphatase; ALB: albumin; GLB: globulin; A/G: albumin/globulin ratio; UA: blood uric acid; WBC: white blood count; ESR: erythrocyte sedimentation rate.

For peer review only

Table 4. Pulmonary function parameters of subjects with pulmonary sarcoidosis, with and without extrapulmonary involvement

Parameter	Isolated pulmonary sarcoidosis	Pulmonary sarcoidosis with extrapulmonary involvement	p-value
VC, L	3.04±0.75	3.09±0.82	0.327
FVC, L	3.03±0.75	3.07±0.81	0.309
FVC % pred	97.5 ± 15.6	96.8 ± 17.2	0.305
FEV1, L	3.03±0.75	3.07±0.81	0.21
FEV1 % pred	88.61 ± 15.08	88.89 ± 16.30	0.452
FEV1/FVC	78.62±7.74	78.94±8.52	0.072
RV, L	1.84±0.43	1.83±0.55	0.317
TLC, L	4.86±0.93	4.89±1.03	0.972
RV/TLC	38.51 ± 7.66	37.73 ± 9.16	0.137
DLCO, ml/min/mmHg	21.1 ± 4.8	20.6 ± 5.2	0.487
Airflow limitation, No. (%)	160 (42.3)	109 (42.2)	0.984
Volume restriction, No. (%)	27 (7.1)	12 (4.7)	0.198

Data are presented as mean±SD or %. VC: vital capacity; FVC: forced vital capacity; FEV1: forced expiratory volume in 1 s; RV: residual volume; TLC: total lung capacity; DLCO: diffusion capacity for carbon monoxide of lung.

For peer review only

Table 5. Factors associated with deteriorating HRCT findings in all subjects according to the logistic regression analysis

Parameter	OR (95% CI)	p-value
Extrapulmonary involvement	0.516（0.232-1.147）	0.104
Corticosteroid therapy	0.941（0.42-2.11）	0.884
FVC% pre	0.998（0.978-1.108）	0.843
FEV1/FVC ratio	0.998（0.954-1.043）	0.928

FVC: forced vital capacity; FEV1: forced expiratory volume in 1 s.

Legend of figures

Figure 1. HRCT findings in sarcoidosis patients. (a) pulmonary micronodules at the time of initial hospitalization; (b) pulmonary involvement during follow-up. (c) enlarged mediastinal lymph node at the time of initial hospitalization; (d) larger mediastinal lymph node during follow-up.

Figure 2. (a) Pulmonary sarcoidosis with or without extrapulmonary involvement; (b) with or without corticosteroid therapy; (c) forced vital capacity (FVC) % predicted; (d) forced expiratory volume in 1 s (FEV1) / FVC ratio of patients who had deteriorating chest radiograph findings and patients who did not have deteriorating chest radiograph findings.

For peer review only

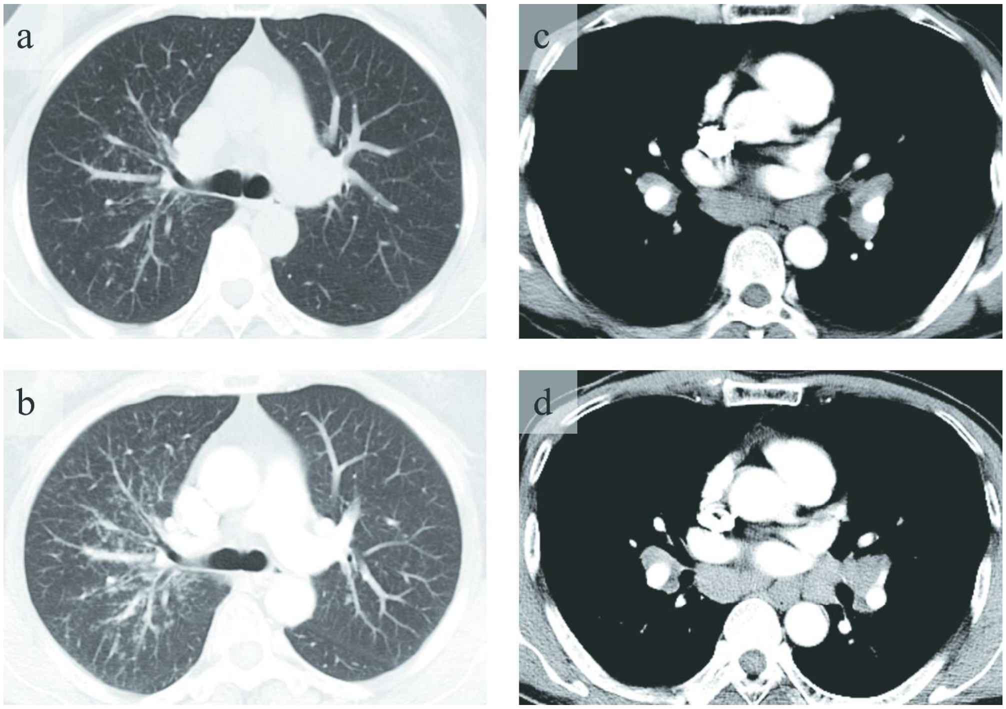

Figure 1. HRCT findings in sarcoidosis patients. (a) Shows pulmonary micronodules at the time of initial hospitalization; (b) with pulmonary involvement during follow-up. (c) Shows enlarged mediastinal lymph node at the time of initial hospitalization; (d) The mediastinal lymph node became larger during follow-up.

268x188mm (300 x 300 DPI)

For peer review only

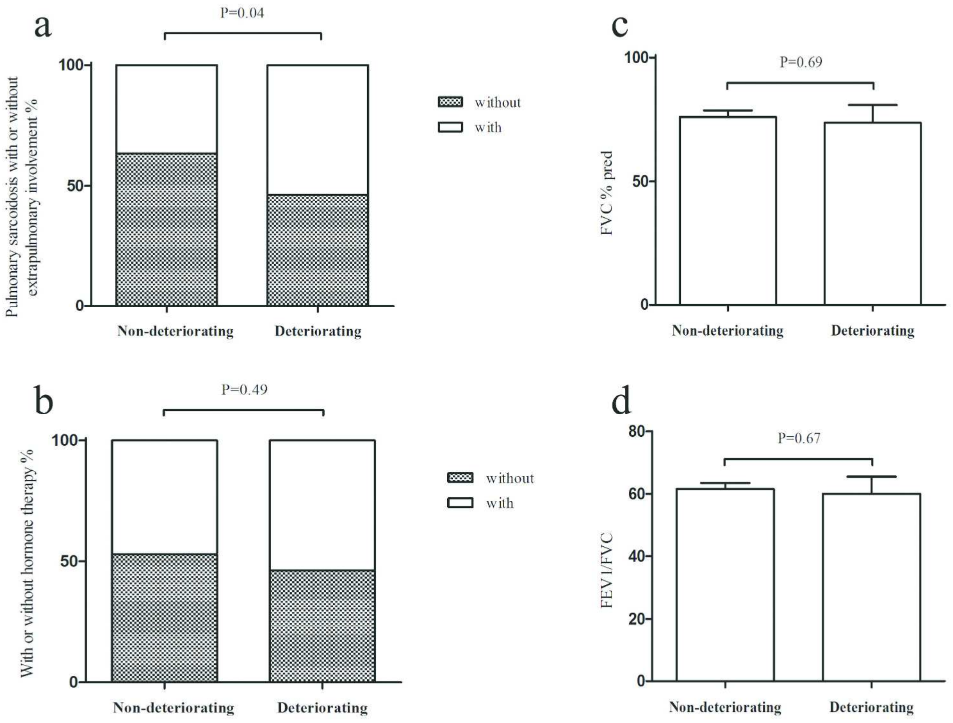

Figure 2. (a) Pulmonary sarcoidosis with or without extrapulmonary involvement; (b) with or without corticosteroid therapy; (c) forced vital capacity (FVC) % predicted; (d) forced expiratory volume in 1 s (FEV1) / FVC ratio of patients who had deteriorating chest radiograph findings and patients who did not have deteriorating chest radiograph findings.

256x190mm (300 x 300 DPI)

STROBE Statement—Checklist of items that should be included in reports of ***cross-sectional studies***

	Item No	Recommendation
**Title and abstract**	1	(*a*) Indicate the study's design with a commonly used term in the title or the abstract P1
		(*b*) Provide in the abstract an informative and balanced summary of what was done and what was found P2
**Introduction**		
Background/rationale	2	Explain the scientific background and rationale for the investigation being reported P4
Objectives	3	State specific objectives, including any prespecified hypotheses P4
**Methods**		
Study design	4	Present key elements of study design early in the paper P4
Setting	5	Describe the setting, locations, and relevant dates, including periods of recruitment, exposure, follow-up, and data collection P4
Participants	6	(*a*) Give the eligibility criteria, and the sources and methods of selection of participants P4
Variables	7	Clearly define all outcomes, exposures, predictors, potential confounders, and effect modifiers. Give diagnostic criteria, if applicable P4
Data sources/ measurement	8*	For each variable of interest, give sources of data and details of methods of assessment (measurement). Describe comparability of assessment methods if there is more than one group P4 P5
Bias	9	Describe any efforts to address potential sources of bias P7
Study size	10	Explain how the study size was arrived at P4
Quantitative variables	11	Explain how quantitative variables were handled in the analyses. If applicable, describe which groupings were chosen and why P4 P5
Statistical methods	12	(*a*) Describe all statistical methods, including those used to control for confounding P5
		(*b*) Describe any methods used to examine subgroups and interactions P5
		(*c*) Explain how missing data were addressed NONE
		(*d*) If applicable, describe analytical methods taking account of sampling strategy P5
		(*e*) Describe any sensitivity analyses P5
**Results**		
Participants	13*	(a) Report numbers of individuals at each stage of study—eg numbers potentially eligible, examined for eligibility, confirmed eligible, included in the study, completing follow-up, and analysed P5 P6
		(b) Give reasons for non-participation at each stage NONE
		(c) Consider use of a flow diagram NONE
Descriptive data	14*	(a) Give characteristics of study participants (eg demographic, clinical, social) and information on exposures and potential confounders P5
		(b) Indicate number of participants with missing data for each variable of interest P5 P6
Outcome data	15*	Report numbers of outcome events or summary measures P5 P6
Main results	16	(*a*) Give unadjusted estimates and, if applicable, confounder-adjusted estimates and their precision (eg, 95% confidence interval). Make clear which confounders were adjusted for and why they were included P6
		(*b*) Report category boundaries when continuous variables were categorized P5 P6

For peer review only

For peer review only - http://bmjopen.bmj.com/site/about/guidelines.xhtml

		(*c*) If relevant, consider translating estimates of relative risk into absolute risk for a meaningful time period P6
Other analyses	17	Report other analyses done—eg analyses of subgroups and interactions, and sensitivity analyses P5 P6
**Discussion**		
Key results	18	Summarise key results with reference to study objectives P6 P7
Limitations	19	Discuss limitations of the study, taking into account sources of potential bias or imprecision. Discuss both direction and magnitude of any potential bias P7
Interpretation	20	Give a cautious overall interpretation of results considering objectives, limitations, multiplicity of analyses, results from similar studies, and other relevant evidence P6 P7
Generalisability	21	Discuss the generalisability (external validity) of the study results P7
**Other information**		
Funding	22	Give the source of funding and the role of the funders for the present study and, if applicable, for the original study on which the present article is based P8

*Give information separately for exposed and unexposed groups.

For peer review only

For peer review only - http://bmjopen.bmj.com/site/about/guidelines.xhtml
